# uPAR-Guided Dendrimer Gel Nanoparticles Reprogram Inflammation to Stabilize Atherosclerotic Plaques

**DOI:** 10.1021/acsami.5c26233

**Published:** 2026-06-24

**Authors:** Hsin-Yin Chuang, Huari Kou, Yue-Wern Huang, Hu Yang

**Affiliations:** † Linda and Bipin Doshi Department of Chemical and Biochemical Engineering, 14717 Missouri University of Science and Technology, Rolla, Missouri 65409, United States; ‡ Department of Biological Sciences, Missouri University of Science and Technology, Rolla, Missouri 65409, United States; § Joint Department of Biomedical Engineering, 5506 Marquette University and Medical College of Wisconsin, Milwaukee, Wisconsin 53226, United States

**Keywords:** PAMAM dendrimer, rapamycin, uPA, atherosclerosis, multi-inlet vortex mixer

## Abstract

Atherosclerosis is characterized by lipid deposition, chronic inflammation, and apoptosis within the arterial wall, leading to plaque progression and instability. Current lipid-lowering therapies fail to fully address residual cardiovascular risk driven by local inflammation and cell death. Here, we report the development of uPA-functionalized, rapamycin-encapsulated dendrimer nanoparticles (G5PM-uPA/RA) that preferentially accumulate in urokinase plasminogen activator receptor (uPAR)-enriched atherosclerotic plaque, including macrophage- and apoptosis-rich lesion microenvironments. G5PM-uPA/RA was constructed by cross-linking reaction-enabled flash nanoprecipitation in a custom-made multi-inlet vortex mixer, followed by thiol-maleimide conjugation of uPA for uPAR-guided targeting. The nanoparticles demonstrated uniform morphology, favorable stability, and efficient rapamycin loading. In vitro, G5PM-uPA/RA exhibited enhanced macrophage uptake (1.3-fold than nontargeted form), sustained intracellular drug retention (2.2-fold than free drug), and effective suppression of inflammatory cytokine TNF-α release (−10%). In vivo biodistribution studies in *Ldlr* ^–/–^ mice confirmed accumulation of G5PM-uPA/RA in aortic lesions. Four weeks of G5PM-uPA/RA treatment in *Ldlr* ^–/–^ mice led to significant reduction in plaque burden (−52% in whole aorta, −41% in aortic root), necrotic core size (−68%), proinflammatory cytokines (−59% for TNF-α, −57% for IL-6), and apoptosis (−61%), while promoting fibrous cap thickening (+60%). Importantly, systemic toxicity was not observed. Collectively, these findings demonstrate that G5PM-uPA/RA offers an effective and safe strategy for inflammation modulation and plaque stabilization, providing a promising nanomedicine platform for atherosclerosis therapy.

## Graphical abstract

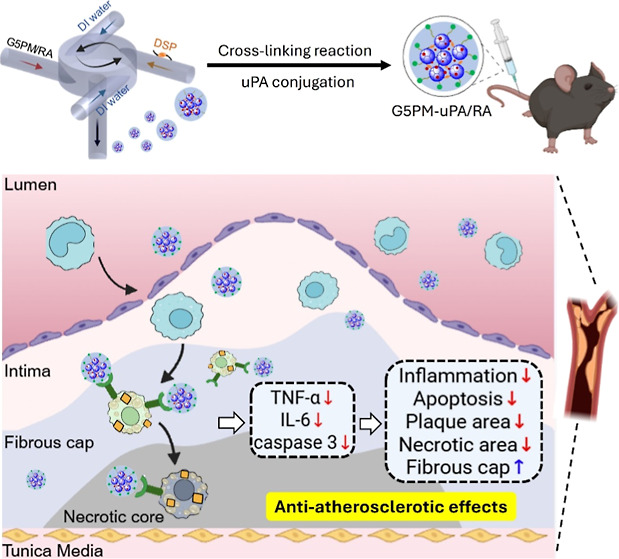

## Introduction

Atherosclerosis remains the leading cause of cardiovascular morbidity and mortality worldwide, characterized by lipid accumulation, chronic inflammation, and cell death within the arterial wall.^1,2^ While statins and PCSK9 inhibitors are commonly used to lower low-density lipoprotein (LDL) levels for atherosclerosis treatment, they do not fully address the residual cardiovascular risk caused by underlying inflammation and apoptosis within atherosclerotic plaques.^3,4^ Excessive inflammatory signaling and apoptosis within plaques driven by macrophage infiltration and cytokine release contribute to necrotic core expansion and thinning of the fibrous cap, thereby destabilizing plaques and increasing the risk of rupture, potentially leading to acute cardiovascular events.^5–7^ Therefore, there is an urgent need to develop novel therapeutic strategies that selectively modulate inflammation in the local plaque microenvironment to halt or reverse atherosclerotic progression.

Inflammation and lipid accumulation within atherosclerotic plaques create a complex and dynamic microenvironment that conventional therapies often fail to address effectively.^8,9^ Nanoparticle-based systems show strong potential to suppress plaque inflammation and attenuate atherosclerosis progression.^10,11^ By enhancing drug solubility, stability, and targeted accumulation in diseased vascular sites, these delivery platforms improve the therapeutic precision.^12,13^ However, heterogeneous plaque composition, inefficient penetration across dysfunctional endothelium, and rapid systemic clearance continue to limit their long-term efficacy. To overcome these barriers, utilizing disease-associated molecular targets offers a promising avenue to enhance the nanoparticle selectivity and retention within plaques.

The urokinase plasminogen activator receptor (uPAR) is plaque-enriched and presents across multiple plaque-associated cell types, including macrophages, endothelial cells, smooth muscle, and apoptotic cells within atherosclerotic lesions, making it an attractive target for site-specific drug delivery.^14,15^ We also confirmed elevated uPAR expression in atherosclerotic lesions of *Ldlr* ^–/–^ mice compared to wild type controls (Figure S1). In our previous study, uPA-mediated dendrimer-based nanoparticles successfully demonstrated selective targeting of uPAR-overexpressing TNBC models, highlighting the strong uPAR-targeting potential of our delivery system for atherosclerosis therapy.^16,17^ Cationic polyamidoamine generation 5 (G5) dendrimers were modified with polyethylene glycol (PEG)-maleimide and further cross-linked with cleavable disulfide linkers (DSP) to obtain G5PM nanoparticles. PEGylation with maleimide groups improves biocompatibility and allows uPA ligand conjugation, while DSP cross-linking enables glutathione-responsive degradation, making G5PM an efficient platform for targeted delivery.

Atherosclerosis is driven by immunometabolic dysfunction, where lipid overload fuels inflammatory/immune signaling and cell death, making therapies that modulate both processes attractive.^18^ While many candidate antiatherosclerotic compounds like flavonoids are under investigation, translation is often limited by poor bioavailability and complex multitarget mechanisms.^19^ Rapamycin (RA), a clinically used mTOR inhibitor, can act across endothelial cells, macrophages, and vascular smooth muscle cells to promote autophagy and suppress inflammatory remodeling, supporting its use for plaque stabilization;^20^ however, systemic rapamycin is limited by off-target metabolic side effects (e.g., dyslipidemia), motivating lesion-targeted delivery that improve lesion-level delivery while limiting systemic burden.^20–22^ Incorporating RA into a uPAR-targeted delivery system may therefore enhance its therapeutic efficacy while minimizing off-target toxicity.^23^

In this study, we developed the novel RA-loaded dendrimer-based nanoparticles (G5PM-uPA/RA) designed to target atherosclerotic plaques through uPA–uPAR interactions (Figure 1a,b). These nanoparticles are able to penetrate the dysfunctional intima, effectively accumulate in plaque lesion, and release rapamycin in a sustained manner. By suppressing proinflammatory cytokines such as tumor necrosis factor-α (TNF-α) and interleukin-6 (IL-6)^24,25^ and reducing caspase-3-mediated apoptosis,^26^ we hypothesized that G5PM-uPA/RA would attenuate plaque burden, limit necrotic core formation, and promote fibrous cap thickening. Collectively, this strategy holds promise for enhancing plaque stabilization and achieving potent antiatherosclerotic effects. Abbreviations used in this study are listed in Table 1.

**Figure 1:**
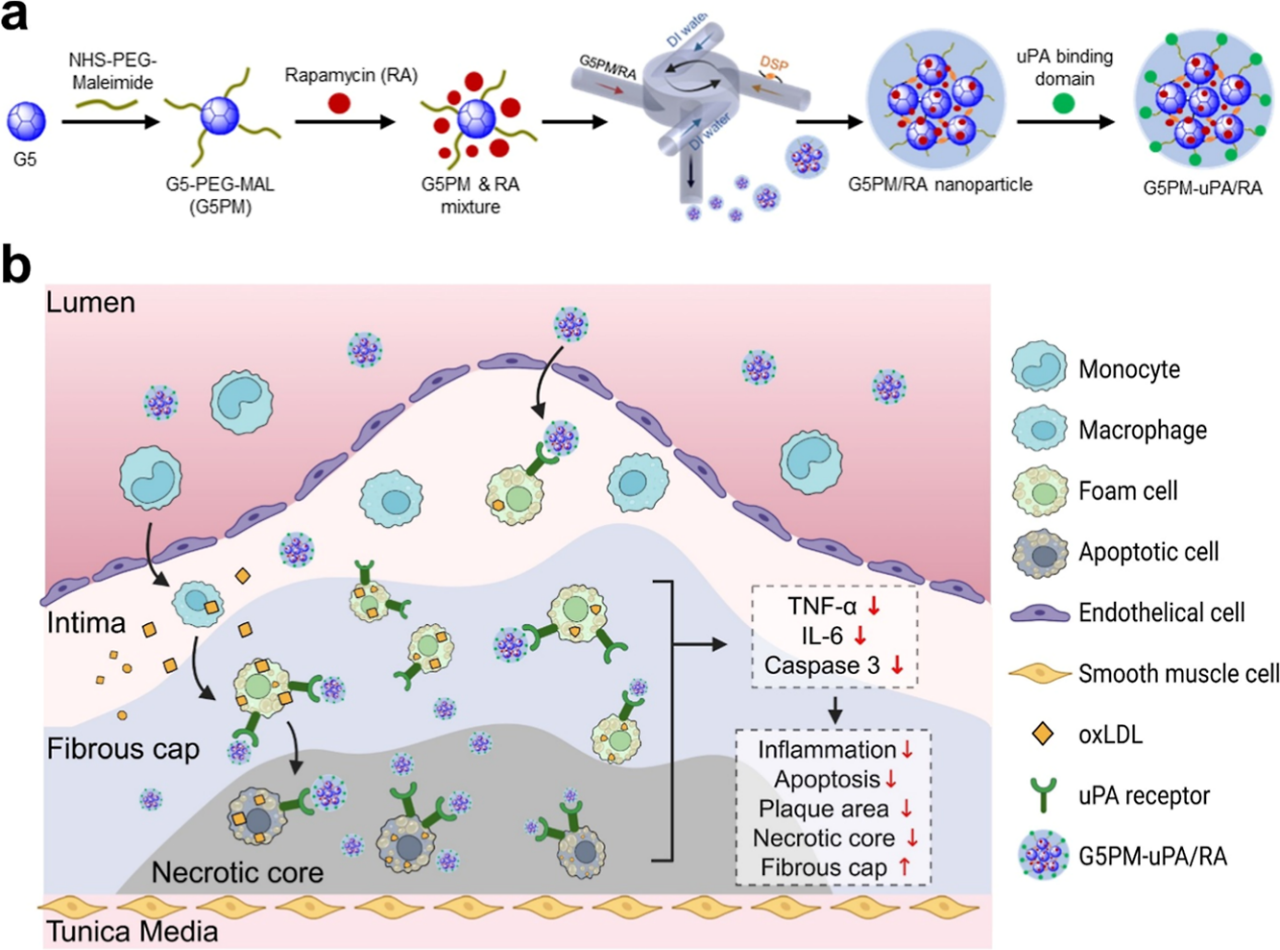
Schematic representation of G5PM-uPA/RA as a potential targeted atherosclerosis therapy. (a) Synthesis process of G5PM-uPA/RA. G5: Polyamidoamine generation 5 dendrimers; RA: rapamycin; DSP: disulfide-containing linker. (b) The proposed distribution and action of G5PM-uPA/RA in an atherosclerotic lesion. G5PM-uPA/RA enter the intima and actively target foam cells and apoptotic cells via uPA–uPAR interaction. Rapamycin release reduces proinflammatory cytokines (TNF-α and IL-6) and apoptotic marker (cleaved-caspase 3), leading to decreased inflammation and apoptosis, smaller plaque area, reduced necrotic core, and increased fibrous cap thickness.

**Table 1:** Abbreviations Used in This Study

abbreviation	definition
uPA	urokinase-type plasminogen activator
uPAR	urokinase-type plasminogen activator receptor
RA	rapamycin
PAMAM	polyamidoamine
G5	generation 5 polyamidoamine dendrimer
DSP	3,3′-dithiodipropionic acid di(*N*-succinimidyl ester)
PEG	polyethylene glycol
NHS	*N*-hydroxysuccinimide
MIVM	multi-inlet vortex mixer
G5PM	G5-DSP-PEG-maleimide dendrimer
G5PM-uPA	uPA-conjugated G5PM dendrimer
G5PM/RA	rapamycin-loaded G5PM nanoparticle
G5PM-uPA/RA	rapamycin-loaded uPA-conjugated G5PM nanoparticle
LC-MS	liquid chromatography–mass spectrometry
EE	encapsulation efficiency
LC	loading capacity
TEM	transmission electron microscopy
DLS	dynamic light scattering
PDI	polydispersity index
DMEM	Dulbecco’s Modified Eagle’s Medium
PBS	phosphate-buffered saline
DMSO	dimethyl sulfoxide
*Ldlr* ^–/–^	low-density lipoprotein receptor knockout
ORO	Oil Red O
MT	Masson’s trichrome
H&E	hematoxylin and eosin
LPS	lipopolysaccharide
DAPI	4′,6-diamidino-2-phenylindole
IRDye800cw	near-infrared dye (NHS ester)
FITC	fluorescein isothiocyanate
OCT	optimal cutting temperature compound
TNF-α	tumor necrosis factor-α
IL-6	interleukin-6

## Materials and Methods

### Materials

Disulfide-containing linker (3,3′-dithiodipropionic acid-di­(*N*-succinimidyl ester), DSP) was synthesized in our lab.^27^ Polyamidoamine generation 5 dendrimer (PAMAM G5) was purchased from Nanosynthons (Mt. Pleasant, MI, USA). NHS-PEG [MW = 3400 g mol^–1^] and NHS-PEG-Maleimide [MW = 3497 g mol^–1^] were obtained from RuixiBiotech Co., Ltd. (Xi’ An City, Shaanxi, China). The murine uPA binding domain (GCQNGGVCVSYKYFSRIRRCS, 21 amino acids)^28^ was synthesized by LifeTein (Somerset, NJ, USA). Rapamycin (RA) was purchased from Thermo Fisher Scientific (Waltham, MA, USA). IRDye800cw NHS ester was acquired from LICORbio (Lincoln, NE, USA).

### Cell Lines and Cell Culture

The RAW 264.7 cell line was purchased from American Type Culture Collection (ATCC, Manassas, VA, USA). The cell line was maintained with Dulbecco’s modified Eagle’s medium (DMEM, from ATCC) supplemented with 10% fetal calf serum (FCS) at 37 °C and 5% CO_2_. The cell culture reagents described above were obtained from Cytiva (Marlborough, MA, USA). Cells were passaged every three to five days in flasks.

### Animals

Four-week-old low-density lipoprotein receptor knockout (*Ldlr* ^–/–^) mice and C57BL/6 wild type mice were purchased from the Jackson Laboratory (Bar Harbor, ME, USA) and housed/bred at the S&T vivarium (Rolla, MO, USA). The mice were maintained in microisolated cages on ventilated racks in a 12 h light/dark cycle in a temperature-controlled room (18–22 °C) and given free access to food and water. Four-week-old *Ldlr* ^–/–^ mice were fed with high-fat diet for 16 weeks to establish an atherosclerosis model.^29,30^ Both male and female mice were used in all in vivo studies, with approximately equal numbers of males and females in each experimental group. The feeding diet was switched to chow diet at week 16 for following in vivo studies. All animal care and use followed the instructions and regulations in an IACUC protocol approved by the Institutional Animal Care and Use Committee of Missouri University of Science and Technology (IACUC reference No. 190-22).

### Synthesis of Functionalized Dendrimer Particles

Two types of dendrimer particles were synthesized: cross-linked PAMAM G5 (G5-DSP-PEG-Maleimide; G5PM; the nontargeted form) and uPA-functionalized cross-linked PAMAM G5 (G5-DSP-PEG-Maleimide-uPA; G5PM-uPA; the targeted form).

To synthesize the G5PM nanoparticle, PAMAM G5 was dissolved in a 0.2 mM NaHCO_3_ solution with a final concentration of 20 mg/mL. NHS-PEG-Maleimide (3400 g mol^–1^) was dissolved in deionized (DI) water with a concentration of 23.4 mg/mL. Equal volumes of both solutions were mixed and stirred at room temperature overnight, allowing the reaction between *N*-hydroxysuccinimide ester and the amine group.^31^ The mixture was then dialyzed against DI water using 3.5 kDa dialysis tubing (Repligen, Boston, MA, USA). DI water was changed every 6–12 h three times. After freeze-drying, the G5PM was obtained as a white powder and stored at −20 °C for later flash nanoprecipitation.

To prepare fluorescently labeled nanoparticles for uptake and biodistribution studies, we dissolved G5PM in DI water (1 mg/mL). For FITC labeling, fluorescein isothiocyanate (FITC) was dissolved in DMSO (5 mg/mL) and added to the G5PM solution at a molar ratio of 1:1 (FITC/G5), followed by stirring at room temperature for 12 h. For near-infrared labeling, IRDye 800cw-NHS (1 mg/mL, aqueous) was mixed with the G5PM solution (1 mg/mL) at a molar ratio of 1:10 (IRDye800cw-NHS/G5) and stirred at room temperature for 12 h. Labeled products were purified by dialysis and collected after freeze-drying, as described above.

As reported in our previous studies,^27,32,33^ a custom-designed four-inlet vortex mixer (MIVM) was used for the synthesis of nanoparticles via flash nanoprecipitation.^34,35^ The MIVM contains four inlet channels and a central mixing chamber; rapid impinging-jet mixing at the chamber induces fast solvent exchange and nanoprecipitation under continuous-flow conditions, improving batch-to-batch reproducibility. One inlet was fed with an aqueous solution of G5PM (1 mg/mL), another with a solution of DSP (6.4 μg/mL) in acetone, and the remaining two with DI water. The pump pushed the four solutions into the MIVM at a rate of 40 mL/min. The product solution was treated on a rotary evaporator to remove acetone. The final product, G5PM nanoparticle, was obtained after dialysis and freeze-drying as described above. To synthesize G5PM-uPA nanoparticles, an appropriate amount of G5PM nanoparticle was dissolved in DI water and then mixed with uPA solution in DI water to ensure a 1:1 molar ratio of PEG-Maleimide and uPA. The mixture was incubated for 15 min to allow conjugation through a thiol-maleimide click reaction.^36^

### Preparation and Characterization of Nanocomplexes

Two types of nanocomplexes were prepared: G5PM-uPA nanoparticle carrying rapamycin (G5PM-uPA/RA; the targeted form, the final complex in this study) and G5PM nanoparticle carrying rapamycin (G5PM/RA; the nontargeted form).

G5PM-uPA/RA was synthesized following the route shown in Figure 1a. RA was loaded by replacing the G5PM solution with a mixture of G5PM (1 mg/mL) and RA (1.2 mg/mL), which was premixed and stirred at room temperature for 12 h. A similar cross-linking procedure was performed, as described previously, to synthesize the G5PM/RA nanocomplex using the mixture of G5PM and RA and DSP in acetone solution, via MIVM. Then, an Amicon Ultra-15 centrifugal filter unit was used to remove acetone and free drug. The G5PM/RA nanocomplex was obtained after being freeze-dried. The G5PM-uPA/RA nanocomplex was prepared using a similar procedure with the addition of a uPA functionalization step. Freeze-dried G5PM/RA nanoparticles were reconstituted in DI water and mixed with uPA solution at a 1:10 molar ratio of G5PM to uPA, which corresponds to a 1:1 molar ratio of PEG-maleimide to uPA. The mixture was incubated at room temperature for 15 min under gentle mixing to allow conjugation via thiol-maleimide click chemistry, forming the final nanocomplex G5PM-uPA/RA. This conjugation ratio and reaction conditions were selected based on our previous publication,^16^ where the same design achieved efficient uPAR-mediated targeting and provided supporting evidence for improved targeting performance.

Size distribution and zeta potential of G5PM-uPA/RA were measured with dynamic light scattering (DLS, Malvern Panalytical Zetasizer Ultra) at 25 °C. Transmission Electron Microscopy (TEM; JEOL JEM-1400) was performed to determine morphology of the final product G5PM-uPA/RA in DI water. Encapsulation efficiency (EE) and loading capacity (LC) of the formulations were determined using liquid chromatography–mass spectrometry (LC–MS; Shimadzu 2020 Single Quadrupole LC/MS). EE (%) and LC (%) were calculated using the following formulas:

$$encapsulation\;efficiency\;\left(EE,\%\right)=\frac{weight\;of\;RA\;in\;final\;product}{weight\;of\;RA\;added}\times 100\%$$

$$loading\;capacity\;\left(LC,\%\right)=\frac{weight\;of\;RA\;in\;final\;product}{weight\;of\;final\;product}\times 100\%$$

### LC-MS Method for RA Quantification

RA analysis was performed using a Shimadzu LCMS-2020 Single Quadrupole LC/MS system. Two mobile phases were used: phase A consisted of 0.1% formic acid in ultrapure water, and phase B consisted of 0.1% formic acid in methanol. Separation was achieved via HPLC using a Hydro-RP column (4 μm, 150 × 2 mm; Phenomenex, Torrance, CA, USA), with a binary flow rate of 0.3 mL/min under the following gradient program: 70% mobile phase B for 0.01 min, followed by a linear gradient from 70% to 95% B between 1.0 and 3.0 min, then from 95% back to 70% B between 3.0 and 5.0 min, and finally held at 70% B from 5.0 to 8.0 min. The column oven temperature was maintained at 40 °C, and the injection volume was 20 μL.

Detection and quantification of eluted analytes were performed using selective ion monitoring (SIM+) mode (936.40 *m*/*z*). The interface temperature was set to 350 °C, and the interface voltage was 4500 V. The chromatogram (Figure S2a) and standard curve (Figure S2b) of RA, obtained by LC-MS, are provided in the Supporting Information.

### MTT Assay

The cytotoxicity of G5PM and G5PM-uPA was determined using the thiazolyl blue tetrazolium bromide (MTT) assay (Bio-Techne, Minneapolis, MN, USA). RAW 264.7 cells were seeded at a density of 1 × 10^4^ cells/well in a 96-well plate and incubated overnight. Cells were then treated with various concentrations (0–50 μg/mL) of G5PM or G5PM-uPA for 24 h. Following treatment, 20 μL of MTT solution (final concentration: 0.5 mg/mL) was added to each well and incubated at 37 °C for 2 h. After incubation, the medium and MTT solution were removed, and 200 μL of DMSO was added to each well to dissolve the resulting formazan crystals. The optical density (OD) at 570 nm was measured using a microplate reader. Cell viability was normalized with the PBS-treated control group.

### Annexin V/7-AAD Assay

To complement the MTT assay and directly evaluate macrophage cytotoxicity, apoptosis was assessed by Annexin V/7-AAD staining and flow cytometry. RAW 264.7 macrophages were seeded in 6-well plates (3 × 10^5^ cells/well) in 2 mL of complete medium and incubated overnight. Cells were then treated with PBS, G5PM, or G5PM-uPA at the same nanoparticle concentration (50 μg/mL) used in the uptake/viability studies for 6 or 24 h. After treatment, cells were collected, washed with PBS, and stained with Annexin V-FITC and 7-AAD. Flow cytometry data were acquired from 1 × 10^4^ single-cell events per sample and analyzed using CytExpert software (Beckman Coulter). Quadrant boundaries were defined using fluorescence-minus-one (FMO) controls for each channel (Annexin V-FITC only and 7-AAD only) together with the unstained control. Gates were applied identically across all treatment groups. Cells were classified as viable (Annexin V^–^/7-AAD^–^), early apoptotic (Annexin V^+^/7-AAD^–^), late apoptotic (Annexin V^+^/7-AAD^+^), or necrotic (Annexin V^–^/7-AAD^+^), and results were reported as the percentage of total cells in each population.

### Cellular Uptake Studies

Flow cytometry, confocal microscopy, and LC-MS analysis were performed to assess cellular uptake. To evaluate the uptake of G5PM and G5PM-uPA, RAW 264.7 cells (3 × 10^5^ cells/well) were seeded in a 6-well plate and incubated 24 h. Cells were treated with PBS, G5PM, or G5PM-uPA (both labeled with FITC) for an additional 6 h. After treatment, all samples were harvested, washed three times with PBS, and analyzed using a CytoFLEX Flow Cytometer (Beckman Coulter, Indianapolis, IN, USA).

Forward scatter height (FSC-H) and side scatter height (SSC-H) were used to gate the main cell population (Gate 1), followed by gating single cells using FSC-H versus forward scatter area (FSC-A) (Gate 2). For each sample, 1 × 10^4^ single-cell events were acquired, and FITC-positive cells were identified by applying a fluorescence threshold in the FITC channel (Ex/Em = 488/525 nm), defined using the PBS control group; this gate was used to quantify the percentage of FITC-positive population (%), and the mean fluorescence intensity (MFI) was calculated for the gated FITC-positive population.

To visualize the cellular uptake of G5PM and G5PM-uPA, RAW 264.7 cells (3 × 10^4^ cells/well) were seeded in an 8-well chambered coverglass system and incubated for 24 h. Cells were then treated with PBS (control), G5PM, or G5PM-uPA (both labeled with FITC, Ex/Em = 491/516 nm) for 6 or 24 h. After treatment, cells were stained with Hoechst33342 (1 μg/mL, Ex/Em = 350/461 nm) at room temperature for 5 min. Samples were washed three times with PBS and imaged using a Nikon A1R confocal microscope and its NIS-Elements AR Software using DAPI (Ex/Em = 405/425–475 nm), FITC (Ex/Em = 488/500–550 nm), and Cy5 (Ex/Em = 640/633–673 nm) channels.

To quantify the cellular uptake of RA, RAW 264.7 cells (3 × 10^5^ cells/well) were seeded in 6-well plates for 24 h. The medium was replaced with RA (5 μg/mL), G5PM/RA, or G5PM-uPA/RA (50 μg/mL, equivalent to 5 μg/mL RA) in a fresh medium. The cells were rinsed with PBS and harvested at various posttreatment time points: 1, 4, 8, 12, and 24 h. The collected samples were diluted and homogenized using a blender with ZrO beads in 0.4 mL of methanol. After centrifugation at 5000*g* for 2 min, the supernatant was filtered using PES 3K filters (VWR, Radnor, PA) to separate encapsulated RA from the nanocomplex. The RA concentrations in the filtrates were subsequently quantified using LC-MS.

### In Vitro Inflammation Study

To evaluate the impact of the nanocomplexes on the inflammatory level in vitro, enzyme-linked immunosorbent assay (ELISA) was performed. RAW 264.7 cells (3 × 10^5^ cells/well) were seeded in 6-well plates for 24 h, followed by pretreatment of lipopolysaccharide (LPS) for 2 h prior to nanocomplex treatment to induce an inflammatory response.^37^ LPS stimulation was limited to 2 h to model acute macrophage activation and early inflammatory signaling rather than prolonged inflammatory cytotoxicity. The medium was then replaced with RA (5 μg/mL), G5PM/RA, or G5PM-uPA/RA (each at 50 μg/mL, RA equivalent to 5 μg/mL RA), and cells were incubated for an additional 24 h. After treatment, the conditioned medium was collected and centrifuged at 1000*g* for 5 min to remove cellular debris. The supernatant was used to quantify TNF-α levels using ELISA kits (R&D Systems, Minneapolis, MN, USA). The cells in each well were harvested, and cell viability was assessed using trypan blue. TNF-α secretion was further normalized to the viable cell number.

### Biodistribution Study and Plaque Targeting Effect Evaluation

The timeline of biodistribution studies in atherosclerotic mice is shown in Figure 4a. Atherosclerotic mice were randomly divided into groups and received different treatments (IRDye800cw, G5PM, G5PM-uPA; *n* = 3/group) by intravenous (IV) injection into the lateral tail vein. G5PM and G5PM-uPA (200 μg/30 g mouse) were labeled with IRDye800cw (Ex/Em = 745/810 nm) as described in the earlier section. IRDye800cw in PBS served as the control group. At 24 h postinjection, the mice were euthanized, and the major organs and aorta were harvested. The biodistribution of G5PM and G5PM-uPA was assessed using the AMI HTX imaging system (Tucson, AZ, USA).

To further investigate the biodistribution of G5PM and G5PM-uPA in C57BL/6 wild type mice, IRDye800cw, G5PM, and G5PM-uPA (200 μg; *n* = 3/group) were administered via IV injection. Mice were euthanized at various time points (2, 6, 24, and 48 h) postinjection. Major organs were harvested and imaged using the AMI HTX system. Results are provided in Supporting Information (Figures S8 and S9).

### Atherosclerotic Lesions Analysis

The timeline of in vivo studies is shown in Figure 5a. After 16 weeks of high-fat diet (HFD) feeding, atherosclerotic mice were switched to a standard chow diet and randomly divided into five groups: baseline, PBS, RA (40 μg per 30 g mouse), G5PM/RA (440–570 μg per 30 g mouse; equivalent to 40 μg RA per 30 g mice), and G5PM-uPA/RA (440–570 μg per 30 g mouse; equivalent to 40 μg RA per 30 g mice). The baseline group was euthanized immediately after the high-fat diet feeding period, while the remaining groups received intravenous injections of PBS, RA, G5PM/RA, or G5PM-uPA/RA three times per week for 4 weeks. Mice were euthanized and perfused with PBS on the third day after the final injection. Plasma samples were collected at the study end point and analyzed for total cholesterol using the Amplex Red Cholesterol Assay Kit (Thermo Fisher Scientific) and MMP-9 using a commercial ELISA kit (R&D Systems, Minneapolis, MN) according to the manufacturer’s instructions.

Whole aortas were fixed in 10% formalin followed by Oil Red O (ORO) staining for plaque area evaluation. The whole aortas were then cut open longitudinally. Images of aortas were captured using a Nikon stereo microscope SMZ18, and the ratio of lesion area to total aortic area (*A*_lesion_/*A*_total_) was quantified using ImageJ software.

The aortic roots attached to the heart were embedded in optimal cutting temperature (OCT) compound and preserved at −80 °C for histological analysis. Serial 8 μm-thick sections containing all three aortic valves were prepared using a Leica CM1860 cryostat. The sections were stained with ORO to evaluate plaque area percentage (*A*_plaque_/*A*_aortic root_, %), hematoxylin and eosin (H&E) to assess necrotic core percentage (*A*_necrotic core_/*A*_plaque_, %), and Masson’s trichrome (MT) to determine fibrous cap percentage (*A*_fibrous cap_/*A*_plaque_, %). Quantification was performed using ImageJ software.^38,39^

### Evaluation of Inflammation and Apoptosis in Atherosclerotic Lesions

Immunofluorescence staining was performed to evaluate and visualize the inflammatory markers (TNF-α and IL-6) and apoptotic markers (cleaved-caspase 3) in aortic lesion. Aortic root sections were fixed in 10% formalin in PBS for 5 min, followed by permeabilization using PBS with 0.4% Triton X-100 (PBST) for 10 min. Sections were then blocked with 10% donkey serum in PBST for 30 min at room temperature.

Sections were incubated with primary antibody (anti-TNF-α, anti-IL-6, anti-cleaved-caspase 3, Table S1) for 1 h at room temperature and then incubated with anti-rabbit secondary antibody conjugated with Alexa Fluor 647 (Ex/Em = 650/671 nm; Invitrogen, Table S1) in the dark for 1 h at room temperature. Nuclei were counterstained with Hoechst 33342 (1 μg/mL; Ex/Em = 350/465 nm) for 5 min at room temperature. Samples were washed three times with PBST between the steps.

After staining, samples were mounted with a coverslip for imaging. Fluorescent images, including *Z*-stack acquisitions (magnification: 10×; 16 layers; 0.5 μm interval), were captured using a Nikon A1R confocal microscope (Nikon Instruments Inc., Tokyo, Japan) and analyzed with NIS-Elements AR Software. Imaging was performed using the DAPI (Ex/Em = 405/425–475 nm) and Cy5 (Ex/Em = 640/633–673 nm) channels. Three negative-staining controls (polyclonal rabbit IgG isotype control, monoclonal rabbit IgG isotype control, and secondary antibody only using donkey antirabbit Alexa Fluor 647) were used to confirm staining specificity (Figure S14). Isotype controls were matched to the primary antibody types and used at the same IgG concentration as the corresponding primary. Imaging parameters were optimized separately for each marker and then kept identical across treatment groups within that marker. Quantification was performed by tracing only the lesion area (plaque ROI) and measuring the mean fluorescence intensity (MFI) within that traced lesion region using ImageJ.

### Immunofluorescence Colocalization with Plaque Lineage Markers

To obtain a granular assessment of nanoparticle localization within plaques, aortic root sections were processed for immunofluorescence staining and colocalization with lineage markers for endothelial cells (CD31), macrophages (CD68), and smooth muscle cells (α-SMA; antibodies listed in Table S1). Aortic root sections used for colocalization analysis were obtained from the 4 week therapeutic study (timeline in Figure 5a). To enable nanoparticle visualization, the final dose of G5PM-uPA/RA was Cy5-labeled, and aortic roots were collected the next day, embedded in OCT, and cryosectioned. Briefly, sections were fixed, permeabilized, washed, and stained with the corresponding primary antibodies, followed by fluorescent secondary antibodies as applicable, and nuclei were counterstained with Hoechst 33342. Confocal images were acquired using *Z*-stack acquisitions (magnification: 20×; 5 layers; 0.5 μm interval) and captured using a Nikon A1R confocal microscope and analyzed with NIS-Elements AR Software. Imaging was performed using DAPI (Ex/Em = 405/425–475 nm), FITC (Ex/Em = 488/525 nm for lineage markers), and Cy5 (Ex/Em = 640/633–673 nm for G5PM-uPA-Cy5 nanoparticles) channels.

### Safety Evaluation

The safety of the formulation was evaluated by monitoring body weight and performing histological analysis of major organs. The heart, lungs, liver, kidneys, and spleen were harvested and processed for H&E staining according to standard protocols.

### Statistical Analysis

All values are presented as mean ± standard deviation (SD). Data normality was assessed using the Shapiro–Wilk test, and homogeneity of variance was evaluated using Levene’s test. For comparisons among multiple groups, statistical tests were selected based on these assumptions: (i) for normally distributed data with equal variance, one-way ANOVA followed by Tukey’s post hoc multiple-comparison tests was used; (ii) for normally distributed data with unequal variance, Welch’s ANOVA followed by the Games–Howell post hoc test was used; and (iii) for non-normally distributed data, the Kruskal–Wallis test was applied with appropriate post hoc multiple comparisons. Significance was defined as *p* < 0.05. Asterisks indicate significance levels as follows: *p < 0.05, **p < 0.01, and ***p < 0.001. For figures using letter-based group annotations, bars that do not share a letter are significantly different (*p* < 0.05), whereas bars that share at least one letter are not significantly different (e.g., “AB” is not significantly different from “A” or “B,” while “A” and “B” are significantly different).

## Results and Discussion

### Characterization of G5PM-uPA/RA

We synthesized the G5PM-uPA/RA nanoparticles via flash nanoprecipitation using a four-inlet vortex mixer (MIVM), based upon our previous studies (Figure 1a).^27,32,33^ The nanoparticles were evaluated for their therapeutic effects on atherosclerosis (Figure 1b). Various RA loading concentrations (0.4, 0.8, 1.2, and 1.6 mg/mL) were tested to optimize encapsulation efficiency (EE, %) and loading capacity (LC, %) in G5PM-uPA/RA (1.0 mg/mL). Figure S3a shows a shift toward larger particle size distribution (108.9, 123.9, and 156.8 nm) with increasing RA concentration (0.4, 0.8, and 1.2 mg/mL); the highest concentration (1.6 mg/mL, 371.3 nm) exhibited multiple peaks, indicating instability of the complex. LC-MS analysis showed that both EE and LC increased with higher RA input (Figure S3b). A concentration of 1.2 mg/mL was selected, balancing favorable size distribution with acceptable EE (7.1%) and LC (8.2%). Although our EE of RA is lower than that of lipid-based carriers, which can reach 68.8–93.1% of EE, our LC is higher than their 0.6–0.7%.^10,40^ Despite the limitation, our optimized formulation provided reproducible nanoparticle formation, stable morphology, and effective payload retention, which were further validated by its superior in vitro drug delivery and in vivo therapeutic performance shown in later sections. The TEM image confirmed the spherical structure of G5PM-uPA/RA nanoparticles (Figure 2a). The hydrodynamic size of G5PM-uPA/RA was 123.7 nm (PDI = 0.3) assessed by DLS (Figure 2b). The stability of G5PM-uPA/RA was tested by monitoring its particle size in PBS at room temperature, and no significant change was observed over 5 days (Figure 2c). The zeta potential of G5PM-uPA/RA (+10.9 ± 0.8 mV) was similar to that of nontargeted G5PM/RA (+12.2 ± 1.3 mV), indicating that conjugation of the uPA binding domain on the surface had no significant influence on zeta potential (Figure 2d). These moderately positive values suggest favorable colloidal stability and support efficient cellular uptake through electrostatic interactions with negatively charged molecules on the cell membrane. Collectively, the synthesized G5PM-uPA/RA formed a well-defined nanostructure with uniform morphology and size stability.

**Figure 2:**
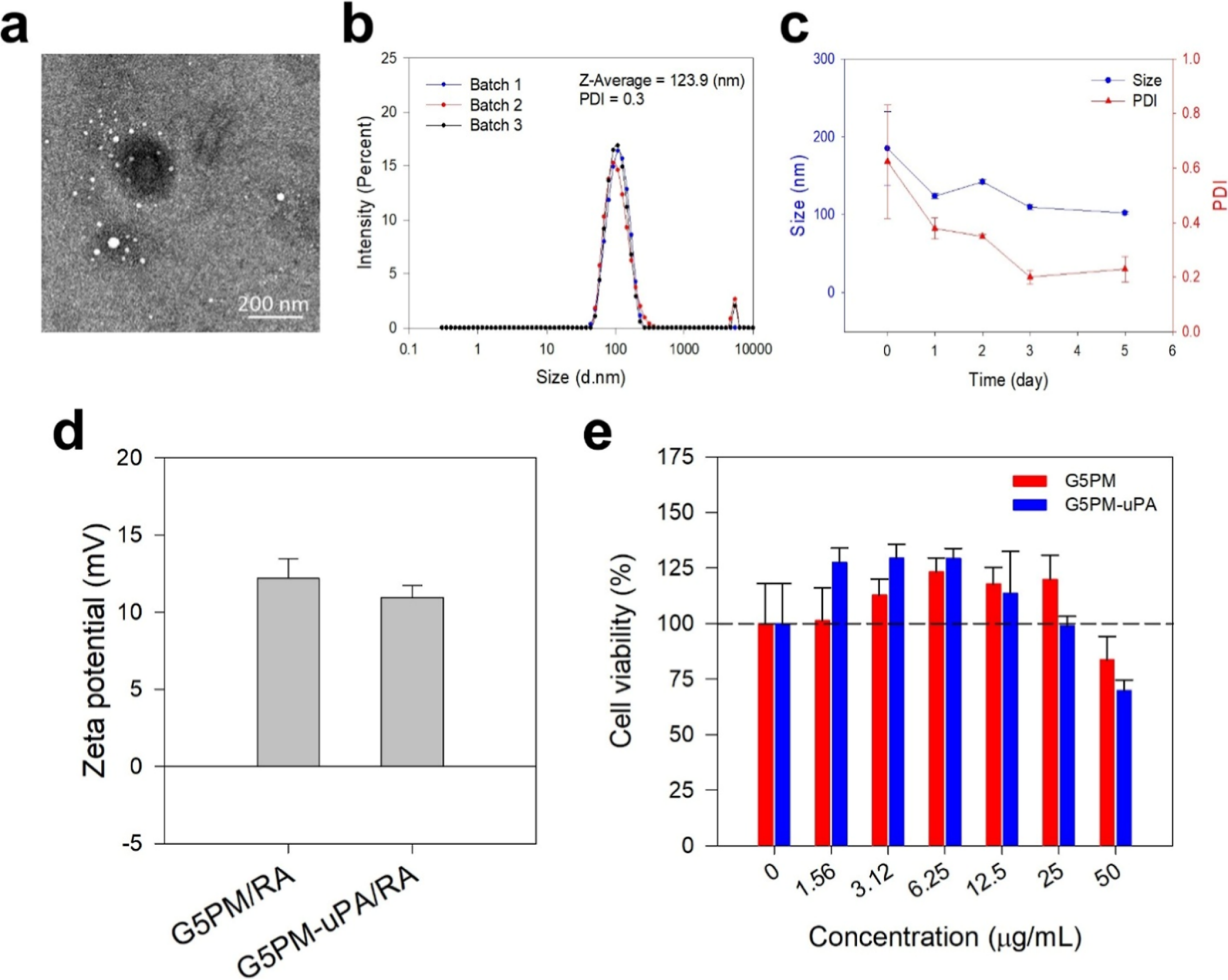
Characterization of G5PM-uPA/RA. (a) TEM image of G5PM-uPA/RA. Scale bar: 200 nm. (b) Hydrodynamic size distribution of G5PM-uPA/RA. (c) Size and polydispersity index (PDI) changes of G5PM-uPA/RA following time. (d) Zeta potential of G5PM-uPA and G5PM-uPA/RA. (e) Cytotoxicity of G5PM and G5PM-uPA in RAW 264.7 cells following 24 h treatment. Data are presented as mean ± SD. *n* = 3 technical replicates per condition. Results are representative of three independent experiments.

### Enhanced Macrophage Uptake and Drug Delivery of G5PM-uPA/RA

MTT results showed that both G5PM and G5PM-uPA nanoparticles exhibited high compatibility (up to 50 μg/mL) in RAW 264.7 macrophage cells after 24 h of treatment (Figure 2e). Consistently, Annexin V/7-AAD analysis indicated no significant difference in viable cell populations (Annexin V^–^/7-AAD^–^) among PBS (74.1 ± 0.5% at 6 h; 78.1 ± 0.2% at 24 h), G5PM (77.7 ± 0.0% at 6 h; 78.2 ± 0.3% at 24 h), and G5PM-uPA (79.3 ± 0.3% at 6 h; 73.3 ± 0.6% at 24 h) groups (Figure S4). At 6 h, no significant differences were observed in early apoptosis, late apoptosis, or necrosis among groups. At 24 h, a statistically significant difference was observed in late apoptosis, with G5PM-uPA (6.7 ± 0.1%, *p* < 0.05) showing a higher late apoptotic fraction than PBS (3.0 ± 0.2%). In addition, necrotic populations at 24 h differed significantly among groups (PBS: 0.4 ± 0.0%; G5PM: 0.7 ± 0.1%, *p* < 0.05 vs PBS; G5PM-uPA: 6.3 ± 0.5%, *p* < 0.01 vs PBS or G5PM), indicating increased cell death in the G5PM-uPA group under these in vitro conditions at 24 h. Together, MTT and Annexin V/7-AAD results indicate generally good short-term compatibility at 6 h, but reveal time-dependent increases in late apoptosis/necrosis at 24 h, which will be considered in future optimization.

Confocal images and flow cytometry were used to analyze both the fraction of cells that internalized FITC-labeled nanoparticles (Figure S5) and the uptake level per cell (Figure 3a,b). At 6 h, both G5PM (89.5 ± 0.6%) and G5PM-uPA (96.4 ± 0.2%) were taken up by the majority of RAW 264.7 macrophages, while G5PM-uPA showed a significantly increased proportion of FITC-positive cells than G5PM (*p* < 0.001). PBS-treated cells were used to define FITC-negative gating (0.3 ± 0.2%, Figure S5). Additionally, G5PM-uPA achieved 1.3-fold higher per-cell uptake than nontargeted G5PM after 6 h of treatment, as reflected by the mean fluorescence intensity (MFI) of the FITC-positive cell population (Figure 3a,b). Similar results were observed after 24 h of treatment (1.3-fold increase in MFI, Figure S6). These results indicate that uPAR targeting increases the proportion of nanoparticle-positive cells and the per-cell nanoparticle uptake.

**Figure 3:**
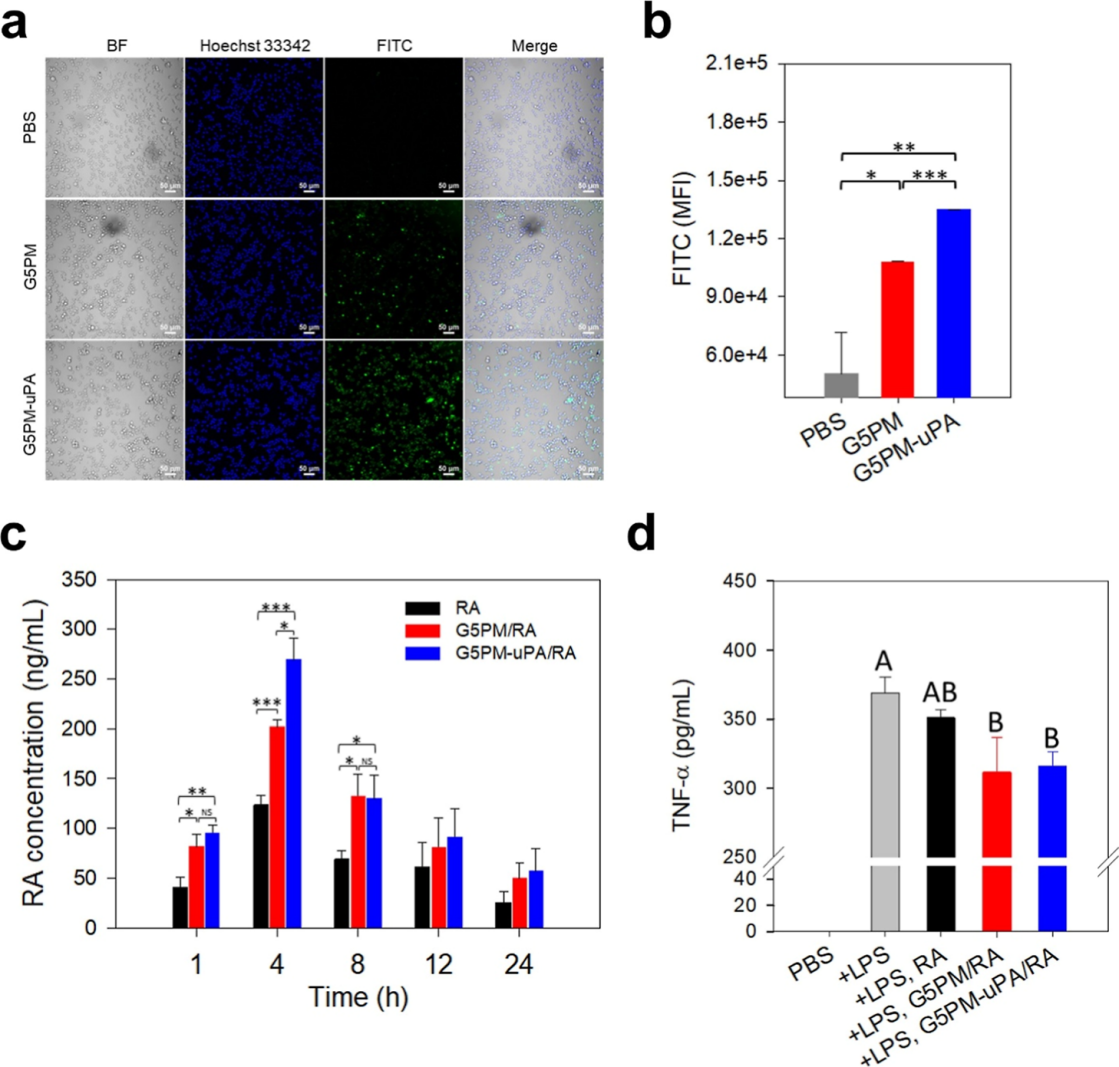
G5PM-uPA/RA improves RA delivery and anti-inflammatory response in RAW 264.7 macrophages. (a) Confocal images and (b) flow cytometry results of after 6 h treatment of FITC-labeled G5PM and G5PM-uPA. Scale bar: 50 μm. Quantification is reported as mean fluorescence intensity (MFI) of gated FITC-positive population. (c) Cellular RA concentrations assessed by LC-MS after treatment of RA, G5PM/RA, and G5PM-uPA/RA at 1, 4, 8, 12, and 24 h (RA equivalent concentration of 5 μg/mL). (d) ELISA analysis for TNF-α levels in the conditioned medium of RAW 264.7 cells treated with RA, G5PM/RA, and G5PM-uPA/RA for 24 h. Cells were pretreated with lipopolysaccharide (LPS) for 2 h to induce inflammatory response. All data are shown as mean ± SD (*n* = 3 technical replicates per condition; results are representative of three independent experiments); *p < 0.05; **p < 0.01; ***p < 0.001; NS: not significant; bars that do not share a letter are significantly different (*p* < 0.05); bars that share at least one letter are not significantly different.

Consistent with this, LC-MS quantification revealed that up to at least 8 h, intracellular RA levels were significantly higher and more sustained in the G5PM-uPA/RA group than in the free RA (2.2-fold at 4 h) or G5PM/RA (1.3-fold at 4 h) groups, and the G5PM/RA group also showed higher RA levels than the free RA group (1.6-fold at 4 h, Figure 3c). To further investigate the anti-inflammatory effects of free drug (RA) and nontargeted (G5PM/RA) and targeted (G5PM-uPA/RA) formulations on macrophages, the conditioned medium of LPS-induced RAW264.7 cells with different treatment was collected for proinflammatory cytokine TNF-α quantification. ELISA analysis demonstrated that both G5PM/RA (311.6 ± 24.9 pg/mL) and G5PM-uPA/RA (316.1 ± 6.0 pg/mL) effectively suppressed LPS-induced TNF-α production (369.0 ± 10.1 pg/mL) after 24 h of treatment, indicating superior anti-inflammatory activity relative to the free RA group (351.0 ± 11.3 pg/mL); interestingly, there was no significant difference between G5PM/RA and G5PM-uPA/RA groups (Figure 3d). These findings indicate that while uPA functionalization enhances cellular uptake and intracellular drug retention, both targeted and nontargeted dendrimer carriers effectively suppress overall TNF-α secretion in RAW 264.7 cells.

When TNF-α secretion was further normalized to the viable cell number (pg/10^6^ cells) to remove the influence of cytotoxicity, a clearer difference between treatment groups was observed (Figure S7). The targeted G5PM-uPA/RA group maintained the lowest TNF-α secretion after normalization (598.6 ± 19.1 pg/10^6^ cells), confirming its strong anti-inflammatory effect (Figure S7b). In contrast, the nontargeted G5PM/RA group showed a significantly increased TNF-α secretion (964.8 ± 77.0 pg/10^6^ cells, *p* < 0.05 vs the control, RA, and G5PM/RA groups), even higher than the control (723.5 ± 22.2 pg/10^6^ cells) and free RA (720.8 ± 12.3 pg/10^6^ cells) groups. Cell viability results (Figure S7a) indicated that G5PM/RA treatment significantly reduced cell viability (63.4 ± 8.9%, *p* < 0.05 vs the control, RA, and G5PM-uPA/RA groups), whereas G5PM-uPA/RA maintained normal viability (103.5 ± 12.8%%, *p* > 0.05 vs the control and RA groups) similar to other groups (control: 100.0 ± 16.1%; RA: 95.4 ± 7.5%). The reduced viability in the G5PM/RA group indicates fewer living cells overall. As a result, the remaining cells showed higher TNF-α levels on a per-cell basis. This observation may result from cell number differences or stress-related secretion, rather than an actual increase in inflammatory signaling. Although both G5PM/RA and G5PM-uPA/RA reduced total TNF-α secretion (pg/mL, Figure 3d), only G5PM-uPA/RA effectively lowered per-cell cytokine production (pg/10^6^ cells, Figure S7b) while maintaining cell viability (Figure S7a). Together, these findings suggest that uPA functionalization of G5PM-uPA/RA enhances anti-inflammatory efficacy with minimal cytotoxicity under the acute LPS stimulation condition, while nontargeted G5PM/RA may trigger stress-related TNF-α release in surviving macrophages.

Despite these encouraging results, one limitation is that our in vitro inflammatory challenge reflects acute LPS stimulation (2 h), which may not fully capture cytotoxicity under prolonged inflammatory stress (e.g., 24 h of LPS exposure). Future work will therefore evaluate nanoparticle effects under extended LPS stimulation in vitro and under a fully atherogenic paradigm with continued high-fat-diet feeding to further assess safety and efficacy in stronger inflammatory conditions.

### Targeting Effects of G5PM-uPA/RA in the Atherosclerotic Mice Model

We next evaluated the targeting capacity of G5PM-uPA in *Ldlr* ^–/–^ mice. IRDye800cw-labeled G5PM or G5PM-uPA were intravenously injected into mice, and major organs (heart, liver, spleen, lung, and kidney) and aortas were harvested 24 h postinjection for ex vivo imaging. Intense fluorescence signals were observed in the liver across all groups, with no significant difference between free dye (3.6 × 10^–5^ photons/s/cm^2^/sr), G5PM (5.1 × 10^–5^ photons/s/cm^2^/sr), and G5PM-uPA (4.6 × 10^–5^ photons/s/cm^2^/sr, Figure 4b,c). Moderate signals were also detected in the kidney (800cw: 2.5 × 10^–5^ photons/s/cm^2^/sr; G5PM: 1.6 × 10^–5^ photons/s/cm^2^/sr; G5PM-uPA: 1.7 × 10^–5^ photons/s/cm^2^/sr) and lung (800cw: 3.8 × 10^–6^ photons/s/cm^2^/sr; G5PM: 4.1 × 10^–6^ photons/s/cm^2^/sr; G5PM-uPA: 1.7 × 10^–5^ photons/s/cm^2^/sr), while the overall biodistribution pattern remained dominated by liver accumulation at 24 h. This biodistribution pattern indicates predominant liver accumulation at 24 h, with additional distribution to the kidney, which is typical for nanoparticle systems whose size ranges from 80 to 200 nm.^41,42^ Importantly, strong fluorescence signals were observed in the aortas (particularly in the aorta root, ascending aorta, and arch of aorta) of the G5PM-uPA group, while only weak signals were detected in the G5PM group and nearly no signal in the free dye group (Figure 4d). This indicates that uPA functionalization markedly enhanced nanoparticle accumulation in atherosclerotic lesions. Notably, no signals were found in the descending thoracic aorta and abdominal aorta in all groups, indicating an early stage of atherosclerosis, which was consistent with our histological results showing plaque developed mainly in the aorta root, ascending aorta, and arch of aorta (Figure 5b,c). Figure 4e shows that the total fluorescence efficiency in the G5PM-uPA group reaches approximately 1.0 × 10^–5^ photons/s/cm^2^/sr, whereas the G5PM and 800cw groups show only about 8.6 × 10^–7^ and 5.4 × 10^–7^ photons/s/cm^2^/sr, respectively. This represents roughly 11.6- and 18.5-fold higher signals for G5PM-uPA compared with the controls, confirming its superior and specific targeting effect on atherosclerotic lesions.

**Figure 4:**
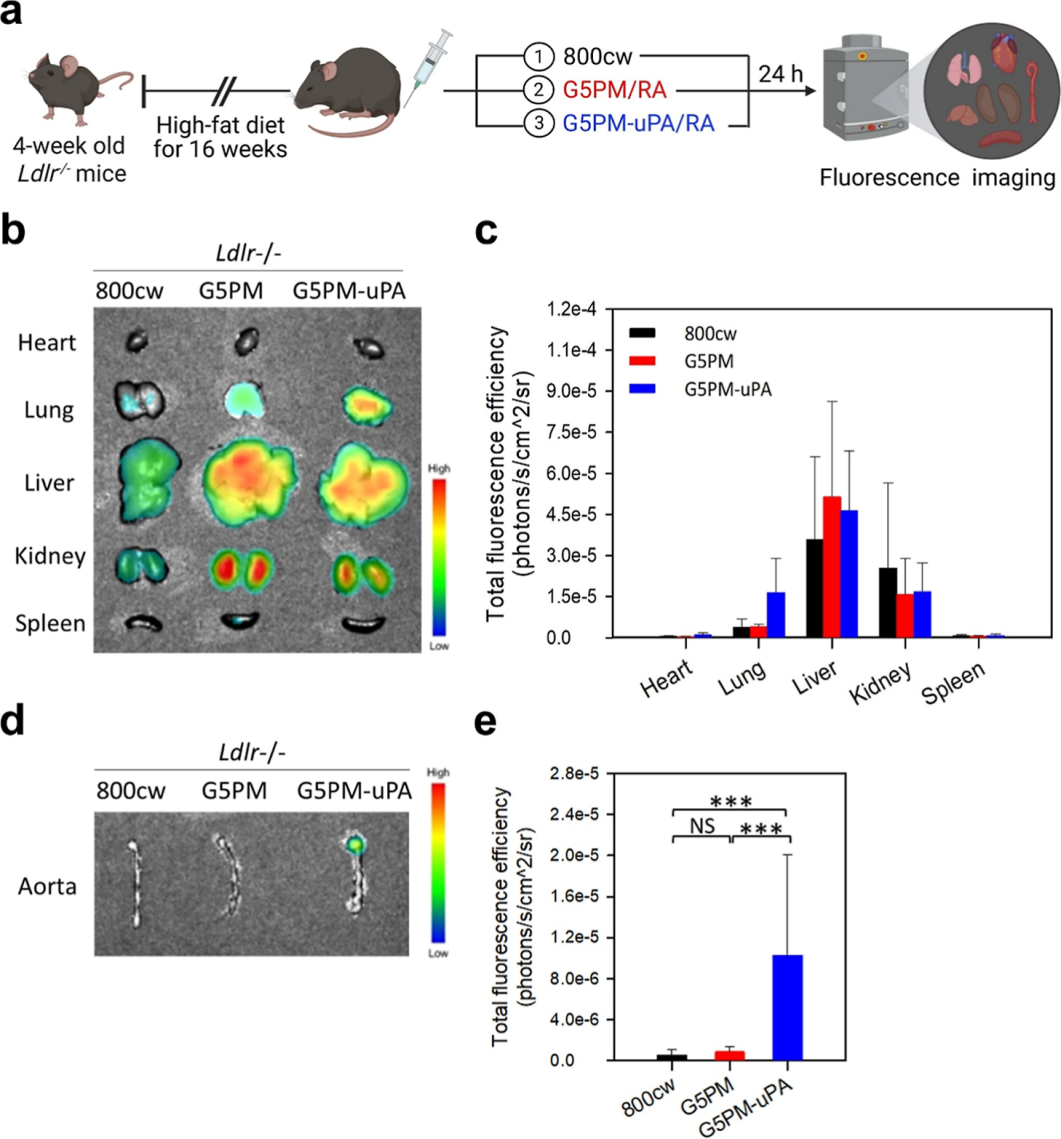
G5PM-uPA shows strong targeting efficacy to aortic plaque in atherosclerosis model mice. (a) Timeline of the biodistribution study and evaluation of plaque-targeting effects. Representative images of the (b) major organs and (d) aortas harvested from the IRDye800cw (800cw), G5PM, and G5PM-uPA groups at 24 h postinjection. G5PM and G5PM-uPA were labeled with IRDye800cw (800cw). Quantified fluorescent intensities in (c) organs and (e) aortas (*n* = 3). All data are shown as mean ± SD (*n* = 3); *p < 0.05; **p < 0.01; ***p < 0.001. NS: not significant (*p* ≥ 0.05).

**Figure 5:**
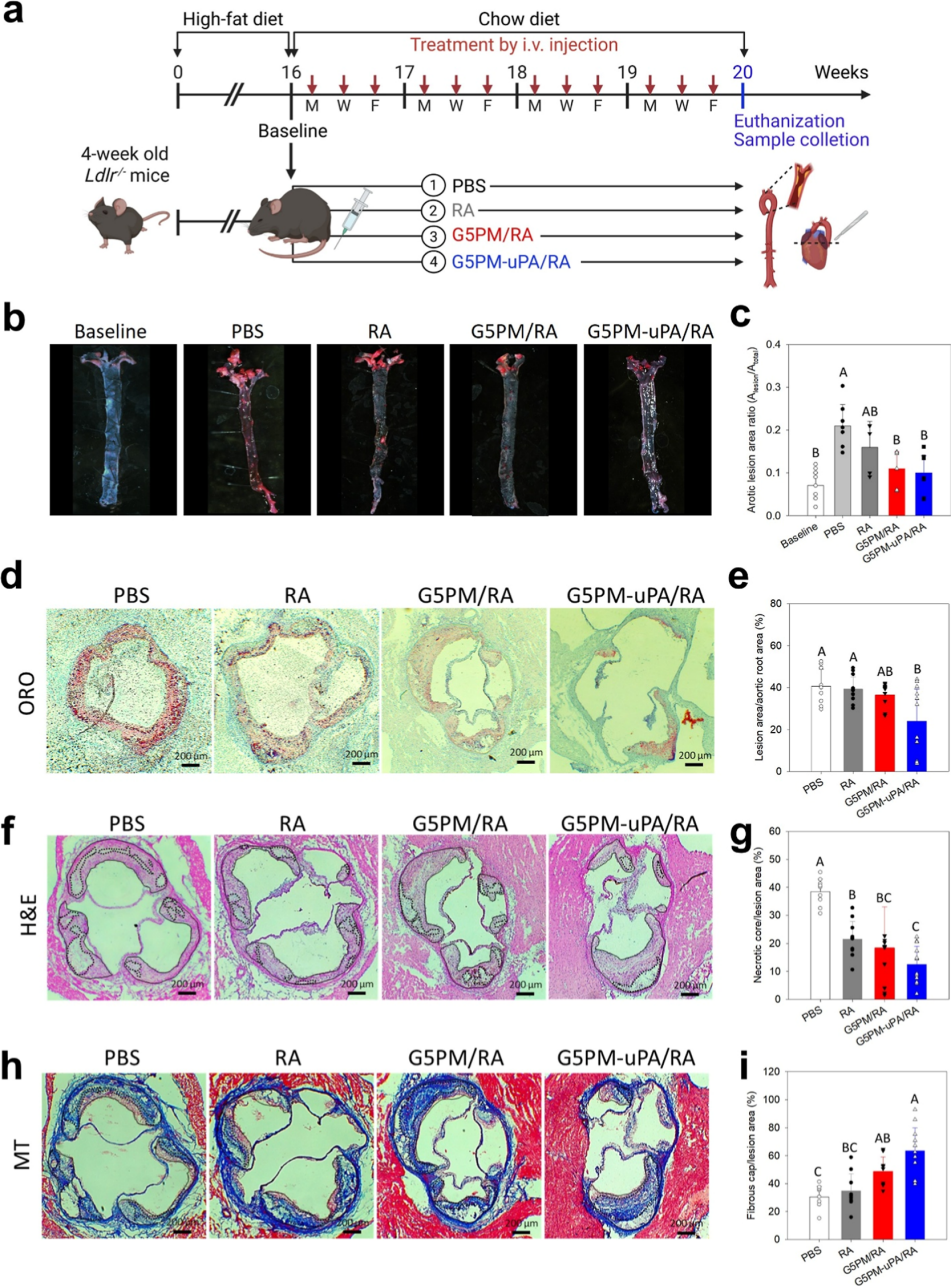
G5PM-uPA/RA shows significant reductions in plaque burden and necrotic core size, while promoting fibrous cap thickening in the atherosclerotic mice model after 4 weeks of treatment. (a) Timeline of atherosclerosis induction and treatment regimen in *Ldlr* ^–/–^ mice. (b) Representative images of aortas and (c) quantitative analysis of the relative plaque area in aortas of the baseline, PBS, RA, G5PM/RA, and G5PM-uPA/RA treatment groups for 4 weeks (*n* = 5–8). (d) ORO staining of the aortic root sections from *Ldlr* ^–/–^ mice with the indicated treatments and (e) corresponding quantitative analysis in the relative plaque area in sections of the aortic root. (f) H&E staining of aortic root sections and (g) quantitative analysis of the necrotic core relative to the plaque area. Solid black lines: plaque areas; black dotted lines: necrotic cores. (h) Masson’s trichrome (MT) staining of aortic root sections and (i) quantitative analysis of the fibrous cap thickness relative to the plaque area. Solid black lines: plaque areas; black dotted lines: fibrous cap areas. Scale bar: 200 μm. Quantitative analysis was performed by ImageJ software (*n* = 3–4, three sections for each mouse). All data are shown as mean ± SD; bars that do not share a letter are significantly different (*p* < 0.05); bars that share at least one letter are not significantly different.

To better define plaque localization at the cellular level, we examined the spatial distribution of G5PM-uPA-Cy5 within atherosclerotic lesions relative to lineage markers for endothelial cells (CD31), macrophages (CD68), and smooth muscle cells (α-SMA; Figure S10). At 24 h postinjection, the G5PM-uPA signal was readily detected within plaque regions and was observed near the luminal surface adjacent to the CD31^+^ endothelium, suggesting access to the plaque–endothelium interface. In addition, the G5PM-uPA signal showed frequent spatial association and partial overlap with CD68^+^ regions within plaques, consistent with uPAR-guided delivery to inflammatory plaque compartments. Because CD68 can also be expressed by lipid-rich intimal smooth muscle cells, these images are interpreted as localization to CD68^+^ plaque regions rather than macrophage-exclusive uptake.^43,44^ Notably, the G5PM-uPA signal was occasionally observed near α-SMA^+^ smooth muscle-rich regions along the fibrous cap/medial interface, but overall showed limited direct overlap, with most nanoparticle fluorescence localized within plaque areas rather than the α-SMA-dense medial layer. This pattern suggests that G5PM-uPA preferentially accumulates in CD68^+^ inflammatory plaque compartments and at the fibrous cap–medial boundary, while deeper penetration into α-SMA^+^ smooth muscle-rich media is more restricted. Overall, these observations indicate that uPAR-guided delivery can access multiple disease-relevant compartments within lesions rather than being strictly macrophage-exclusive, which may be beneficial given emerging evidence that promoting autophagy in smooth muscle cells can contribute to cholesterol removal and plaque stabilization.^45^ Because the fluorescent label was applied to the final dose, these images represent end point localization rather than cumulative distribution across the full treatment course. Additional lineage markers and quantitative colocalization analyses will be pursued in follow-up studies to more precisely define cell-type uptake and mechanisms relevant to plaque stabilization.

The biodistribution of G5PM and G5PM-uPA was also investigated in C57BL/6 wild type mice. At 24 h postinjection, strong fluorescence accumulation was observed mainly in the liver for both G5PM and G5PM-uPA groups, with lower signals detected in the kidneys and other organs (Figure S8). Quantitative analysis confirmed that the fluorescence intensities in the liver and kidneys were the highest, with no significant differences between G5PM (6.5 × 10^–6^ and 1.6 × 10^–6^ photons/s/cm^2^/sr, respectively) and G5PM-uPA (8.2 × 10^–6^ and 1.8 × 10^–6^ photons/s/cm^2^/sr, respectively), indicating similar biodistribution profiles (Figure S8b). This distribution pattern suggests that the nanoparticles showed predominant liver accumulation with a secondary signal in the kidneys, consistent with the biodistribution results observed in *Ldlr* ^–/–^ mice (Figure 4b,c). We further monitored the time-dependent biodistribution of G5PM-uPA in C57BL/6 wild type mice (Figure S9). Rapid accumulation of G5PM-uPA in the liver and kidneys was observed as early as 2 h postinjection, as confirmed by quantitative analysis (Figure S9b). These findings suggest that G5PM-uPA nanoparticles did not exhibit abnormal organ retention, supporting their favorable biodistribution and biocompatibility profiles in wild type mice.^46^

### Antiatherosclerotic Effects of G5PM-uPA/RA in the Atherosclerotic Mice Model

The therapeutic efficacy of G5PM-uPA/RA was evaluated in the *Ldlr* ^*–/–*^ mice model followed by a 4 week treatment regimen (Figure 5a). *Ldlr* ^*–/–*^ mice were fed with a high-fat diet for 16 weeks to develop atherosclerotic plaques. At week 16, the diet was switched to a chow diet, and mice were intravenously injected with different treatments three times a week (on Monday, Wednesday, and Friday) for 4 weeks. Mice were euthanized at week 20 for en face analysis of whole aortas (Figures 5b,c and S11 and Tables S2 and S3) and aortic root cross sections stained with various methods for histological assessment (Figure 5d–i and Tables S2 and S3). Histological improvements, such as reduced necrotic core size and enhanced fibrous cap thickness, are well-established correlates of plaque stabilization and reduced rupture risk.^47–49^

En face analysis of whole aortas revealed extensive plaque burden (red) stained by Oil Red O (ORO) in the PBS group (0.21 ± 0.05, 3.0-fold to the baseline: 0.07 ± 0.03, *p* < 0.001; Figure 5b,c). Notably, this chow-switch phase in our *Ldlr* ^–/–^ model does not necessarily represent a classical regression paradigm; consistent with our previous study using the same regimen, this model can continue to show lesion progression during this 4 week period after diet switch.^27^ The aortic lesion area was modestly reduced by free RA (23.8% lower than the PBS, *p* > 0.05), whereas both G5PM/RA (47.6% lower than the PBS, *p* < 0.01) and G5PM-uPA/RA (52.4% lower than the PBS, *p* < 0.05) treatments significantly decreased the aortic lesion area (Figure 5b,c). No significant difference was observed among RA (0.16 ± 0.06), G5PM/RA (0.11 ± 0.04), and G5PM-uPA/RA (0.1 ± 0.04) groups. The aortic lesion area in the G5PM/RA and G5PM-uPA/RA groups even showed no significant difference from the baseline group (euthanized immediately at week 16), suggesting that both nanoparticle formulations attenuated further lesion progression during the post-high-fat follow-up period. Overall, lesions were predominantly distributed in the ascending and arch parts of the aortas in this model.

Consistent with these findings, ORO staining of aortic root cross sections demonstrated significantly smaller plaque areas (red region) in the G5PM-uPA/RA group (24.0 ± 15.3%) compared with the PBS (40.7 ± 8.2%, *p* < 0.05) and RA (39.3 ± 5.8%, *p* < 0.05) groups but not with G5PM/RA (36.5 ± 5.8%, *p* > 0.05; Figure 5d,e). In addition to the plaque area, we quantified plaque lipid content as the ORO-positive lipid area normalized to the total plaque area (%) in aortic root sections (Figure S12). Compared with PBS (15.0 ± 8.3%), lipid fraction showed no significant difference in RA (9.0 ± 4.7%) and was significantly lower in both nanoparticle-treated groups (G5PM/RA: 5.5 ± 5.4%, *p* < 0.05 vs PBS; G5PM-uPA/RA: 4.0 ± 2.7%, *p* < 0.05 vs PBS), consistent with reduced lipid-rich plaque burden. Histological analysis by H&E staining further showed that G5PM-uPA/RA (12.5 ± 6.5%) significantly reduced the necrotic core area (black dotted lines) relative to the total plaque area (black solid lines) compared with the PBS (38.5 ± 4.6%, *p* < 0.001 vs PBS) and RA (21.6 ± 6.2%, *p* < 0.05) groups, whereas RA (*p* < 0.05 vs PBS) and G5PM/RA (18.4 ± 14.7%, *p* < 0.001 vs PBS) produced significant but less pronounced effects than the PBS (Figure 5f,g).

Moreover, Masson’s trichrome (MT) staining revealed a significant increase in collagen content (black dotted lines, indicating fibrous cap) relative to the total plaque area (black solid lines) in the G5PM-uPA/RA (63.5 ± 16.5%) group compared to the PBS (30.4 ± 7.6%, *p* < 0.001) and RA (34.9 ± 12.3%, *p* < 0.001) groups, indicating enhanced plaque stability (Figure 5h,i). G5PM/RA (48.7 ± 10.3%) showed a significantly increased fibrous cap area than the PBS group but no significant difference compared with the RA and G5PM-uPA/RA groups (*p* > 0.05).

Although both G5PM/RA and G5PM-uPA/RA inhibited plaque progression, only the targeted G5PM-uPA/RA further reduced the size of the necrotic core and increased the thickness of the fibrous cap better than free RA did. Because a portion of the nanoparticles accumulated in the liver (Figure 4b,c), we also measured plasma total cholesterol levels to assess whether treatment effects could be attributed to systemic lipid lowering. Plasma total cholesterol levels measured at the end point did not differ significantly among PBS (213.8 ± 28.0 mg/dL), RA (315.4 ± 37.5 mg/dL), G5PM/RA (230.1 ± 50.9 mg/dL), and G5PM-uPA/RA (272.5 ± 13.2 mg/dL) groups (Figure S13), suggesting that the observed plaque effects were not primarily driven by systemic lipid lowering. Together, these results demonstrate that G5PM-uPA/RA effectively suppresses plaque development and remodels the plaque structure by reducing necrotic core formation and promoting fibrous cap thickening, thereby providing strong antiatherosclerotic benefits.

### Evaluation of Anti-inflammatory and Antiapoptotic Effects of G5PM-uPA/RA in the Atherosclerotic Mice Model

The anti-inflammatory and antiapoptotic effects of G5PM-uPA/RA were further examined in atherosclerotic lesions of *Ldlr* ^–/–^ mice after 4 weeks of treatment (Figures 6 and S14 and Tables S4 and S5). Negative staining controls confirm minimal nonspecific Cy5 signals in aortic root sections. Decreased expression of TNF-α and IL-6 and a reduced cleaved level of caspase-3 are well-known indicators of suppressed inflammation and apoptosis, respectively.^50–52^ Immunofluorescence staining followed by lesion ROI-based ImageJ quantification revealed strong expression of proinflammatory cytokines TNF-α (Figure 6a,b) and IL-6 (Figure 6c,d) in the aortic sections from the PBS group (TNF-α: 1.00 ± 0.34; IL-6:1.00 ± 0.33). In the representative zoomed plaque views, TNF-α and IL-6 signals appeared broadly distributed within the plaque, with prominent signals along the luminal/plaque interface and in cap/shoulder-like regions. Because TNF-α and IL-6 can be produced and released by multiple plaque-resident cell types (e.g., macrophages, smooth muscle cells, and endothelial cells), their immunofluorescence signals may appear broadly distributed across lesions rather than confined to a single microregion, consistent with prior reports.^53–55^ The cleaved caspase-3 signal was primarily detected near the plaque–medial boundary and in regions beneath the necrotic core in the representative images (Figure 6e). As a marker of apoptotic signaling, cleaved caspase-3 can occur in multiple plaque compartments and does not necessarily localize exclusively to the necrotic core,^56^ contributing to a more diffuse lesion-wide staining pattern.^53,57,58^ These signals were only modestly reduced by free RA (TNF-α: 0.64 ± 0.51, *p* > 0.05; IL-6:0.72 ± 0.42, *p* > 0.05) and G5PM/RA (TNF-α: 0.60 ± 0.56, *p* > 0.05; IL-6:0.37 ± 0.31, *p* < 0.05). In contrast, treatment with G5PM-uPA/RA led to significantly greater reductions in both TNF-α (0.41 ± 0.34, *p* < 0.05) and IL-6 (0.43 ± 0.33, *p* < 0.01) levels compared to the PBS group, with no significant difference compared with the RA and G5PM/RA groups. Similarly, cleaved-caspase 3, a key apoptotic marker, was highly expressed in the PBS group (1.00 ± 0.49) but significantly reduced following treatment with free RA (0.50 ± 0.25, *p* < 0.05), G5PM/RA (0.51 ± 0.27, *p* < 0.05), and most notably G5PM-uPA/RA (0.39 ± 0.22, *p* < 0.01; Figure 6e,f). No significant difference was observed among the RA, G5PM/RA, and G5PM-uPA/RA groups. These findings indicate that although free RA has little anti-inflammatory and significant antiapoptotic activities, dendrimer-based encapsulation substantially enhances its bioavailability and functional efficacy in vivo. Furthermore, the superior performance of G5PM-uPA/RA compared to G5PM/RA suggests that uPA-mediated targeting not only improves drug delivery but also enables more consistent modulation of inflammatory and apoptotic signaling within atherosclerotic plaques. The dual anti-inflammatory and antiapoptotic actions of G5PM-uPA/RA highlight its potential as a therapeutic agent for promoting plaque stabilization. Therefore, plasma matrix metalloproteinase-9 (MMP-9) levels were measured at the study end point as a systemic indicator of matrix remodeling associated with plaque instability.^59^ Plasma MMP-9 did not differ significantly among PBS (0.82 ± 0.03 ng/mL), RA (0.76 ± 0.00 ng/mL), G5PM/RA (0.74 ± 0.03 ng/mL), and G5PM-uPA/RA (0.71 ± 0.12 ng/mL) groups after 4 weeks of treatment (Figure S15), suggesting that circulating MMP-9 is not markedly altered under our conditions. Because MMP-9 activity is primarily regulated locally within lesions, future studies will evaluate plaque-local MMP-9 expression/activity together with smooth muscle markers to further define molecular mechanisms of stabilization.

**Figure 6:**
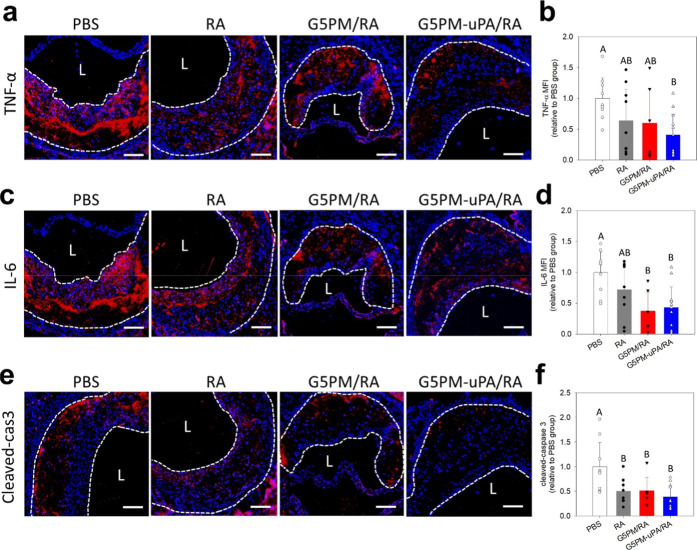
G5PM-uPA/RA significantly reduces inflammation and apoptosis in atherosclerotic plaque after 4 weeks of treatment. Immunofluorescence staining images and quantification of the inflammatory cytokines (a,b) TNF-α and (c,d) IL-6 and apoptotic marker cleaved-caspase 3 (e,f) in sections of the aortic root. Blue: nucleus; red: TNF-α, IL-6, or cleaved-caspase 3; dashed lines outline plaque boundaries; L indicates the vessel lumen. Scale bar: 100 μm. Quantification was performed in ImageJ by manually tracing the lesion region of interest (ROI) and measuring the mean fluorescence intensity (MFI) within the traced lesion ROI (*n* = 3–4 mice per group, three sections per each mouse). All data are shown as mean ± SD; bars that do not share a letter are significantly different (*p* < 0.05); bars that share at least one letter are not significantly different.

### In Vivo Safety Evaluation

Body weight change and histological analysis were conducted to evaluate the safety of G5PM-uPA/RA in *Ldlr* ^–/–^ mice following 4 weeks of treatment. Body weights in all treatment groups declined (ranging from −10.5% to −17.2%) during the administration duration, which was anticipated due to the switch from high-fat diet to chow diet (Figure S16a). No significant differences in body weight changes (%) were observed among all of the treatment groups during the dosing period, suggesting that the observed weight loss was diet-related rather than treatment-induced.

Additionally, no apparent signs of injury or differences were observed in H&E-stained sections of main organs (hearts, lungs, livers, kidneys, and spleens) among all treated groups (Figure S16b), further demonstrating that G5PM-uPA/RA did not introduce additional systemic toxicity beyond the dietary effects. These results suggest that intravenous administration of G5PM-uPA/RA is well-tolerated and safe at the test doses and duration.

Several rapamycin nanodelivery approaches have been explored, including biomimetic leukosomes and ligand-modified liposomes, which can improve vascular targeting but involve higher formulation complexity and variability (e.g., membrane-derived components) and require longer administration duration (up to 8 weeks) to achieve significant reduction in the plaque area compared to free RA.^10,60^ In contrast, our uPAR-guided dendrimer gel nanoparticles are produced by a scalable synthesis and designed to concentrate rapamycin in uPAR-enriched plaques, enabling robust lesion modulation within a shorter treatment window. Consistent with this design, our in vivo data show significantly stronger lesion modulation in 4 weeks without introducing systemic toxicity (Figures 5 and 6 and S16).

## Conclusions

We successfully developed a rapamycin-loaded uPA-functionalized dendrimer nanoparticle (G5PM-uPA/RA) designed for targeted therapy of atherosclerosis. This nanoplatform demonstrated stable morphology, efficient drug encapsulation, and enhanced macrophage uptake in vitro. In the *Ldlr* ^–/–^ mouse model, G5PM-uPA/RA preferentially accumulated in aortic plaques and effectively reduced plaque area, necrotic core size, and proinflammatory cytokine expression, while increasing fibrous cap thickness. Importantly, the treatment induced no additional systemic toxicity, supporting a favorable safety profile. Collectively, these findings highlight the therapeutic potential of G5PM-uPA/RA to achieve both anti-inflammatory and plaque-stabilizing effects, offering a promising strategy to address residual cardiovascular risk beyond conventional lipid-lowering therapies.

## Supplementary Material



## Notes

Immunofluorescent staining of aortic roots, LC-MS chromatogram and standard curve of RA, DLS size distribution of G5PM/RA, encapsulation efficiency and loading capacity of G5PM/RA, Annexin V/7-AAD flow cytometry, FITC-positive population of flow cytometry, macrophage uptake of G5PM/RA and G5PM-uPA/RA, TNF-α secretion normalized to the viable cell number, biodistribution of nanoparticles in wild type mice, time-dependent biodistribution of G5PM-uPA, plaque localization of Cy5-labeled G5PM-uPA and colocalization with vascular lineage markers, Oil Red O-stained aortas, plasma cholesterol levels, negative staining controls, plasma MMP-9 levels, safety evaluation by body weight monitoring and H&E histology of major organs, antibodies used in immunofluorescence, and quantitative and statistical summaries of histological and immunofluorescence results (PDF)
