# Supplementary material for: uPAR-Guided Dendrimer Gel Nanoparticles Reprogram Inflammation to Stabilize Atherosclerotic Plaques
Source: ACS Appl Mater Interfaces. 2026 Jun 24;18(26):36455–69. doi: 10.1021/acsami.5c26233 (PMC13352503; doi:10.1021/acsami.5c26233)
Supplement: Supplementary file 1 [file am5c26233_si_001.pdf]

Supporting Information

uPAR-Guided Dendrimer Gel Nanoparticles Reprogram Inflammation to Stabilize Atherosclerotic Plaques

Hsin-Yin Chuang[1], Huari Kou[1], Yue-Wern Huang[2,*], Hu Yang[3,*]

[1]Linda and Bipin Doshi Department of Chemical and Biochemical Engineering, Missouri University of Science and Technology, Rolla, MO 65409, United States

[2]Department of Biological Sciences, Missouri University of Science and Technology, Rolla, MO 65409, United States

[3]Joint Department of Biomedical Engineering, Marquette University and Medical College of Wisconsin, Milwaukee, WI, United States

*To whom correspondence should be addressed: Dr. Yue-Wern Huang: huangy@mst.edu; Dr. Hu Yang: huyang@mcw.edu

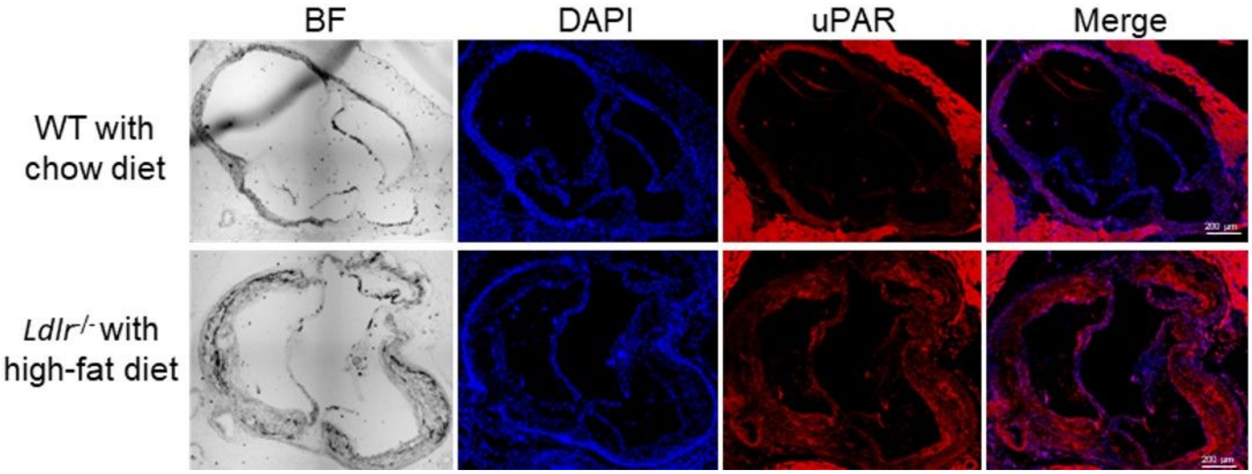

Figure S1. Immunofluorescent images of aortic root from atherosclerosis mice and C57BL/6 wild type mice. High level of uPAR is shown in atherosclerotic plaque. BF: bright field; blue: nucleus; red: uPAR; scale bar: 200 μm.

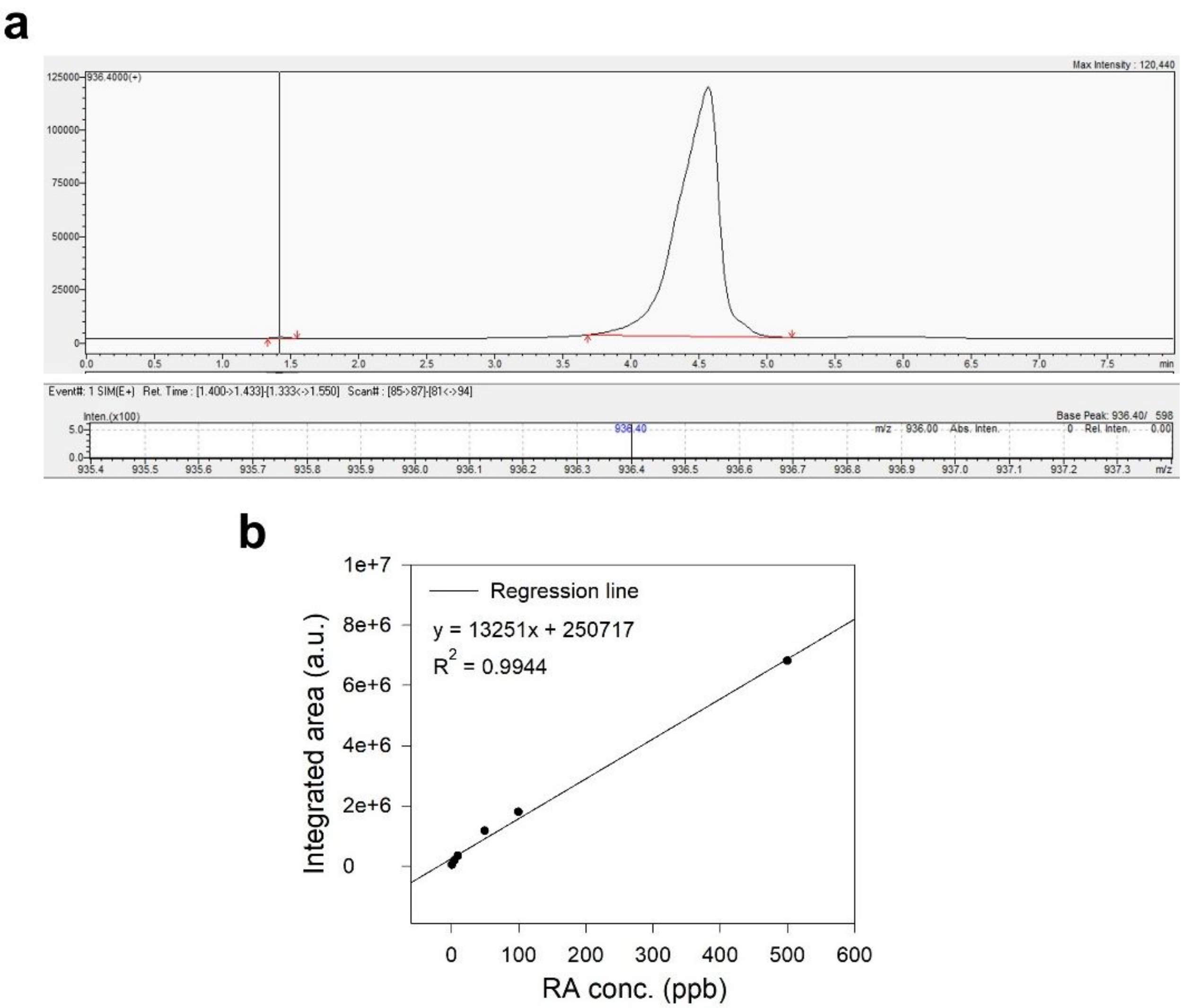

Figure S2. The chromatogram and standard curve of RA accessed by LC−MS. (**a**) Chromatogram of RA (concentration: 500 ppb; 936.40 m/z). (**b**) Standard curve of RA concentration versus integrated area.

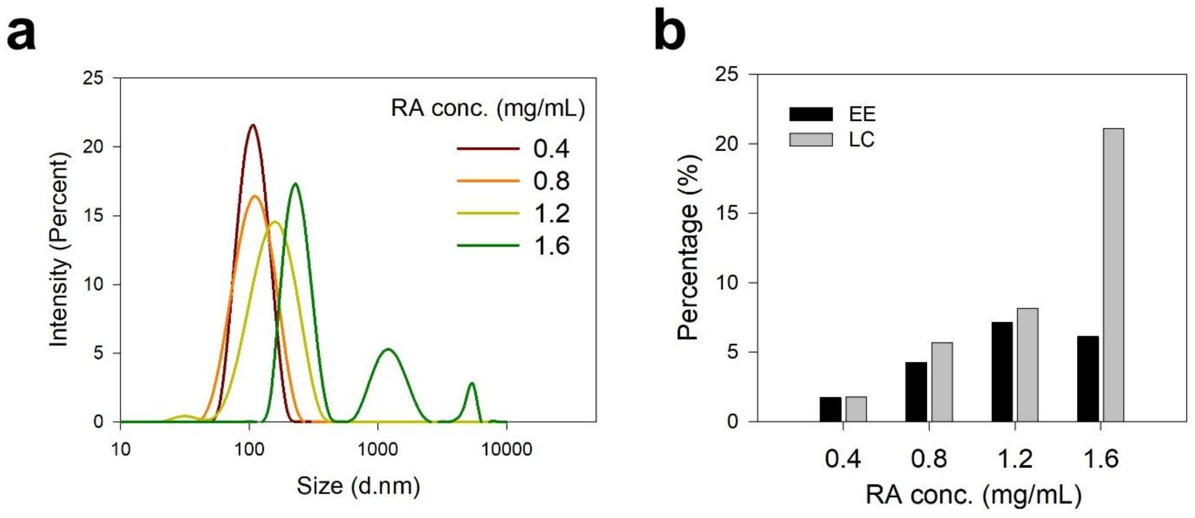

Figure S3. RA encapsulation studies. (**a**) DLS size distributions of prepared G5PM/RA with different RA concentrations (0.4, 0.8, 1.2, 1.6 mg/mL)**.** (**b**) Encapsulation efficiency **(**EE) and loading capacity (LC) of G5PM/RA synthesized with different RA concentrations (0.4, 0.8, 1.2, 1.6 mg/mL; G5PM/RA concentration was fixed at 1.0 mg/mL) determined by LC−MS.

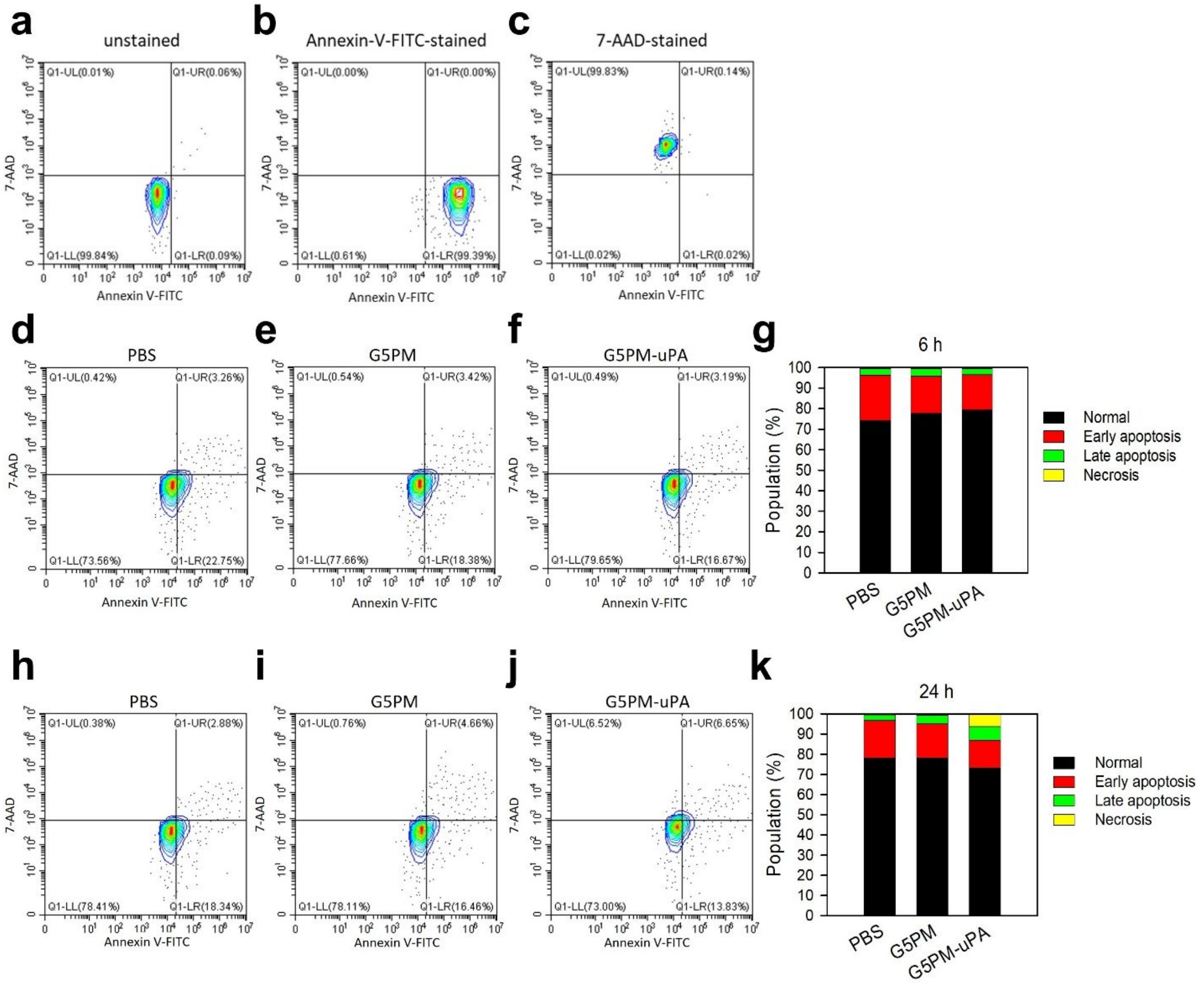

Figure S4. Annexin V/7-AAD flow cytometry confirms minimal macrophage cytotoxicity after nanoparticle treatment. (**a**) Unstained cells, (**b**) Annexin V-FITC single-stained and (**c**) 7-AAD single-stained positive controls used to define quadrant boundaries. Representative four-quadrant plots and quantitative analysis at (**d-g**) 6 h and (**h-k**) 24 h. LL: viable cells (Annexin V^{-}/7-AAD^{-}); LR: early apoptotic (Annexin V^{+}/7-AAD^{-}); UR: late apoptotic (Annexin V^{+}/7-AAD^{+}); and UL: necrotic (Annexin V^{-}/7-AAD^{+}). Stack bar color: black: normal; red: early apoptosis; green: late apoptosis; and yellow: necrosis. Data are shown as mean (n = 3 technical replicates per condition; representative of three independent experiments).

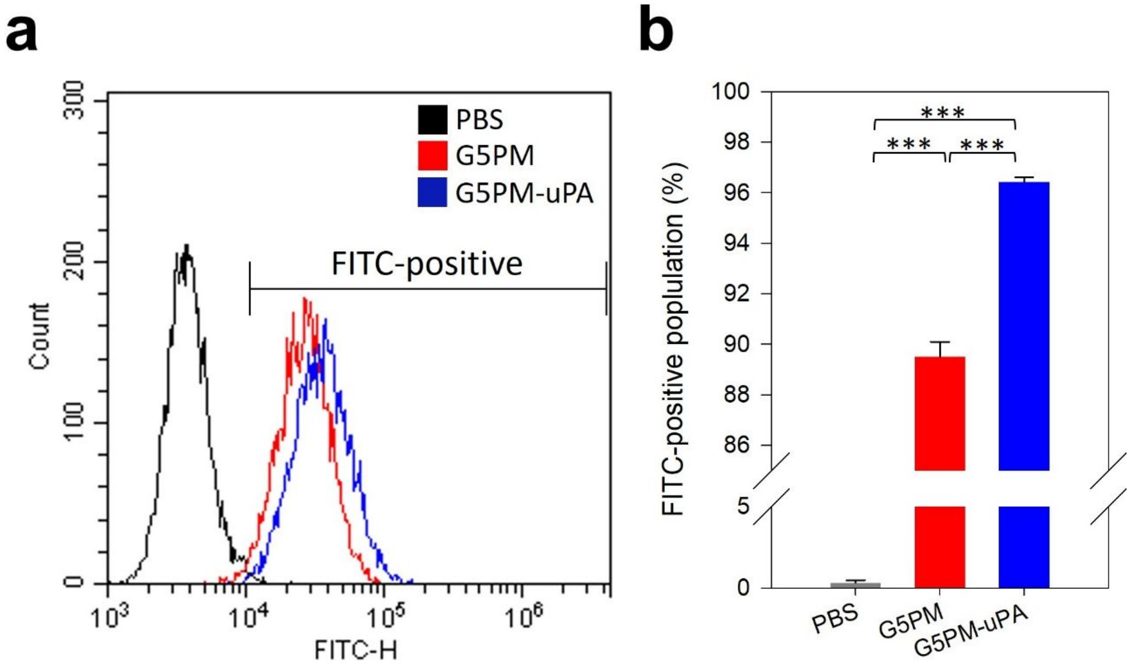

Figure S5. Flow cytometry analysis of RAW 264.7 macrophage uptake of FITC-labeled G5PM and G5PM-uPA at 6 h. (**a**) Representative histogram of FITC-positive gating and (**b**) quantification results of FITC-positive population (%). All data are shown as mean ± SD (n = 3 technical replicates per condition; results are representative of three independent experiments); ***$p < 0.001$.

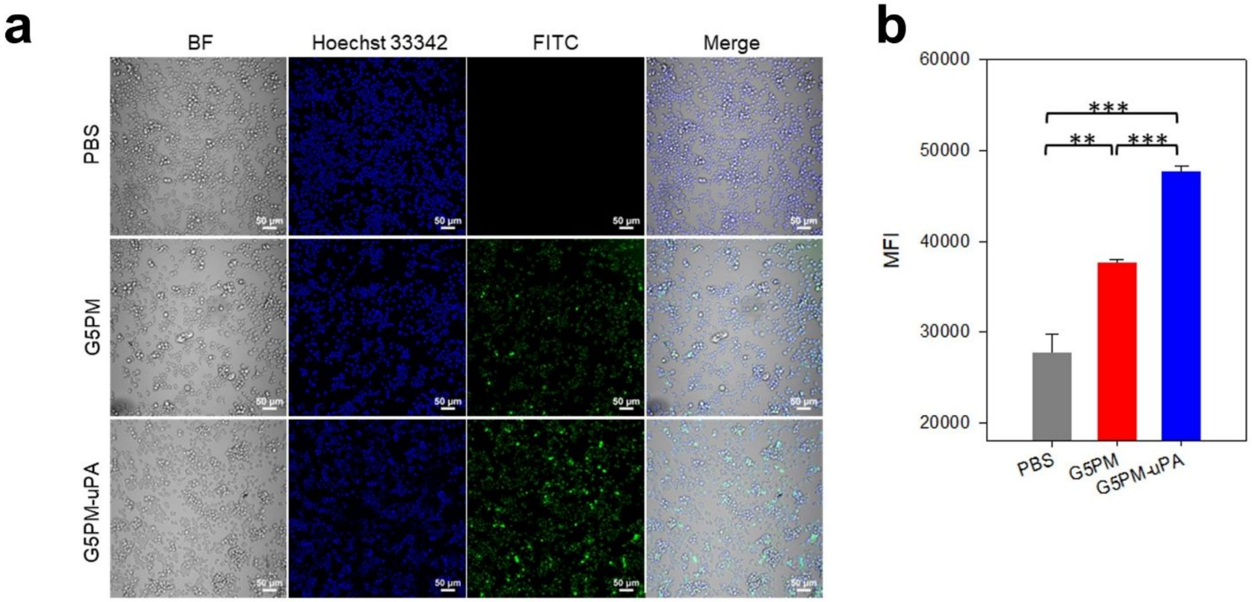

Figure S6. G5PM-uPA shows enhanced cellular uptake in RAW 264.7 cells compared to the PBS and G5PM after 24-h treatment. (**a**) Confocal images and (**b**) flow cytometry results (n = 3). All data are shown as mean ± SD (n = 3 technical replicates per condition; results are representative of three independent experiments); *$p < 0.05$; **$p < 0.01$; ***$p < 0.001$.

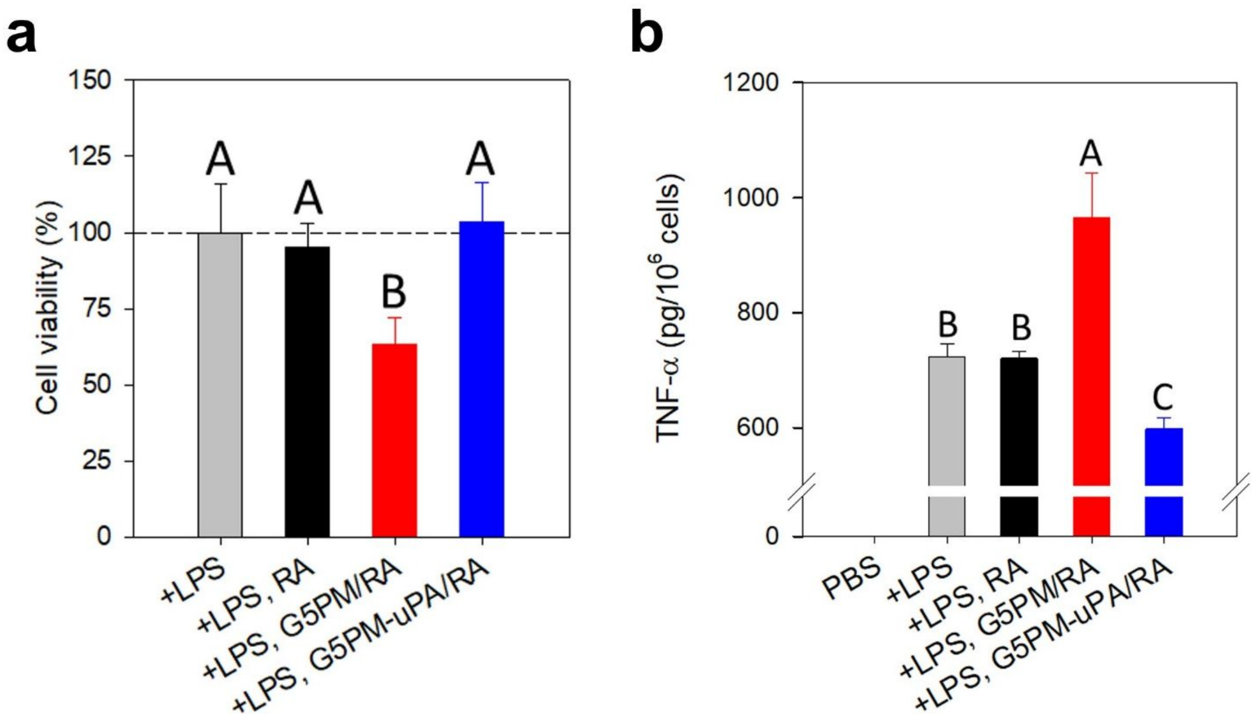

Figure S7. TNF-α secretion normalized to viable cell number (pg/10^6 cells) in lipopolysaccharide (LPS)-stimulated RAW 264.7 macrophages after 24 h treatment. (**a**) Cell viability assessed by trypan blue and (**b**) ELISA analysis normalized to viable cell number. uPA functionalization not only enhances anti-inflammatory effects but also attenuates cytotoxicity observed in non-targeted nanoparticles. All data are shown as mean ± SD (n = 3 technical replicates per condition; results are representative of three independent experiments); bars that do not share a letter are significantly different ($p < 0.05$); bars that share at least one letter are not significantly different.

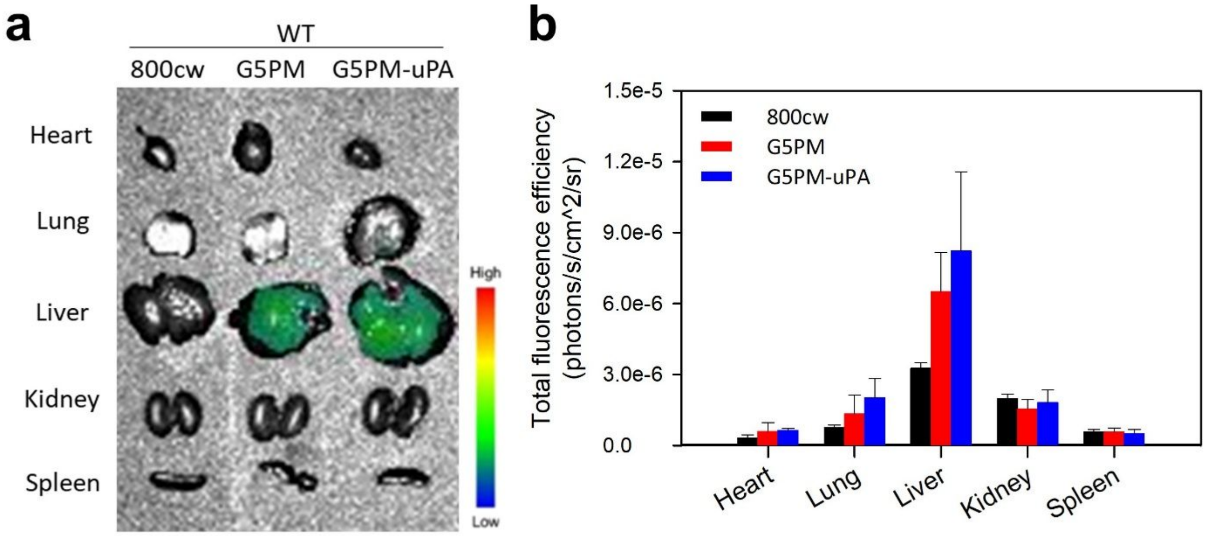

Figure S8. No significant difference is observed among biodistributions of free dye 800cw, G5PM and G5PM-uPA in C57BL/6 wild type mice, indicating similar clearance behaviors. (**a**) Representative images and (**b**) quantified fluorescent intensities of major organs from IRDye800cw, G5PM, and G5PM-uPA groups at 24 h postinjection (n = 3) in C57BL/6 wild type mice.

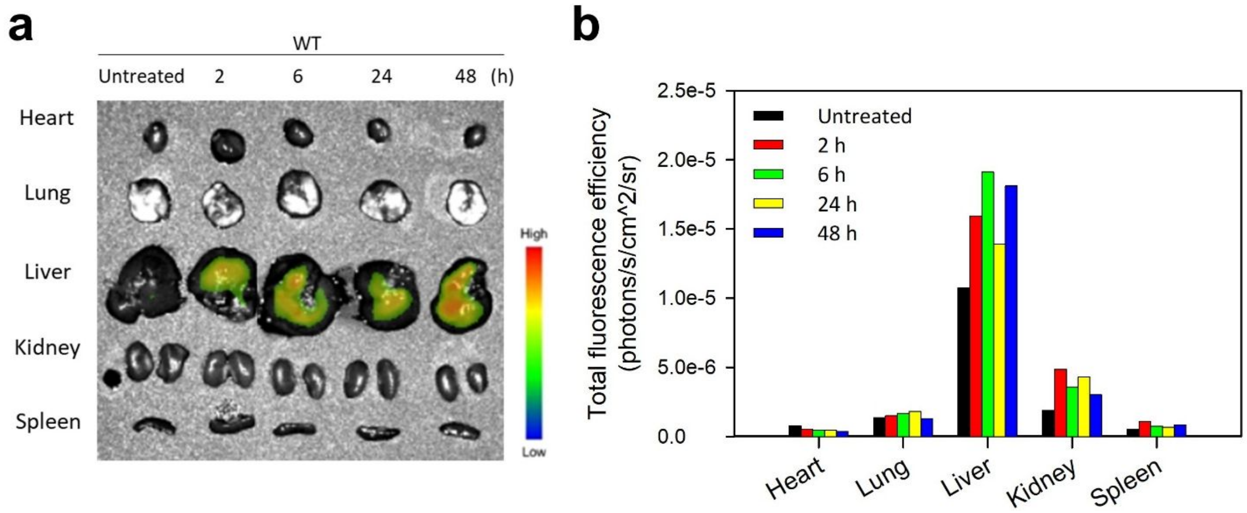

Figure S9. Rapid accumulation of G5PM-uPA in the liver and kidneys in C57BL/6 wild type mice, indicating hepatic and renal clearance routes without abnormal organ retention. (**a**) Fluorescent image and (**b**) quantified fluorescent intensities of main organs at 2, 6, 24, 48 h post-injections.

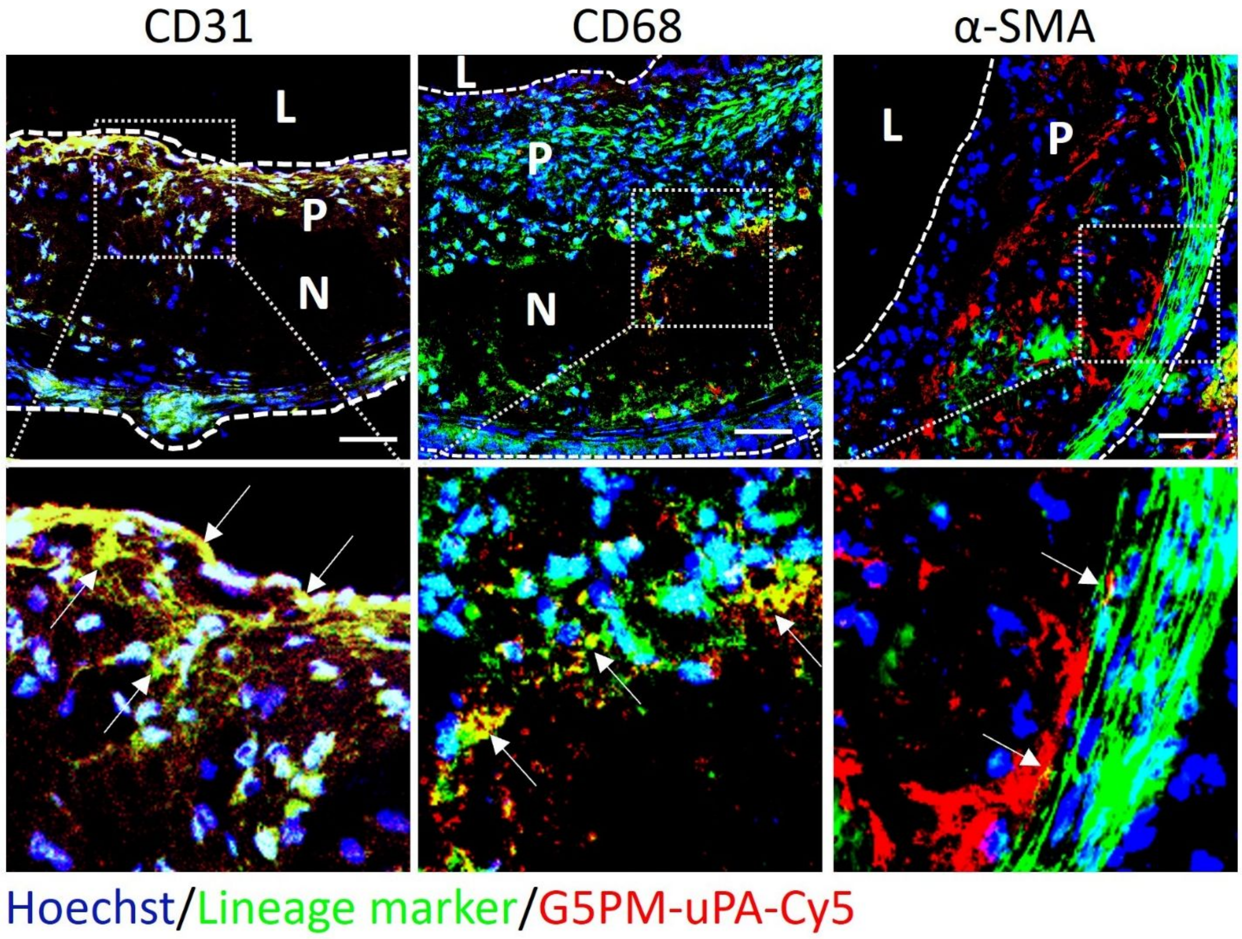

Figure S10. Plaque localization of Cy5-labeled G5PM-uPA and colocalization with vascular lineage markers. Representative confocal immunofluorescence images of atherosclerotic aortic root sections showing G5PM-uPA-Cy5 signal (red) relative to plaque-associated lineage markers (green): CD31 (endothelial cells), CD68 ($CD68^+$ plaque cell populations such as macrophages and lipid-rich smooth muscle cells), and α-SMA (smooth muscle cells). Nuclei are stained with Hoechst 33342 (blue). Dashed outlines indicate vessel boundaries; L, lumen; P, plaque; N, necrotic core. Bottom panels show higher-magnification views of boxed regions. Scale bar: 60 µm. Arrows: colocalization of lineage marker and G5PM-uPA-Cy5 (yellow). Results are representative of three independent experiments.

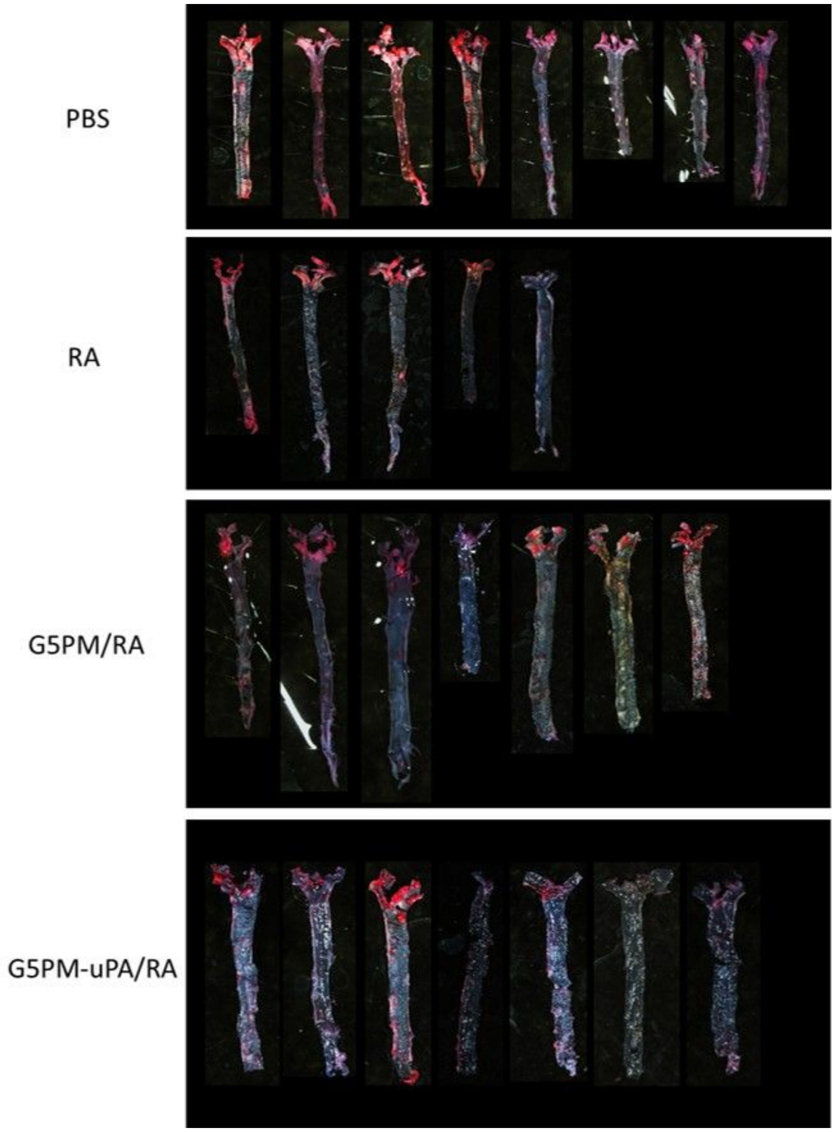

Figure S11. ORO-stained aorta images of different treatment groups. Representative image of each group is included in Figure 5b.

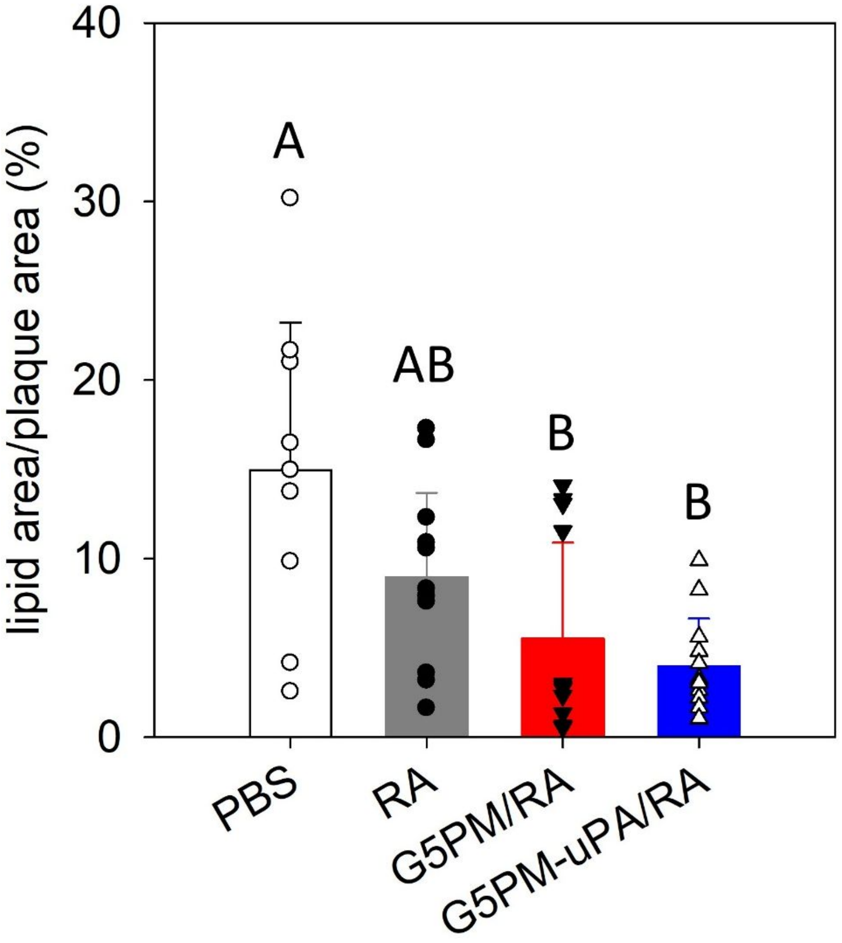

Figure S12. Plaque lipid content quantified from ORO-stained aortic root sections. Oil Red O (ORO) staining was used to quantify lipid-rich area within aortic root plaques. Plaque lipid content was calculated as ORO-positive lipid area normalized to total plaque area (%) for each section. Data are shown as mean ± SD (PBS: 15.0 ± 8.3%; RA: 9.0 ± 4.7%; G5PM/RA: 5.5 ± 5.4%; G5PM-uPA/RA: 4.0 ± 2.7%). Quantitative analysis was performed by ImageJ software (n = 3~4, three sections for each mouse). All data are shown as mean ± SD; bars that do not share a letter are significantly different ($p < 0.05$); bars that share at least one letter are not significantly different.

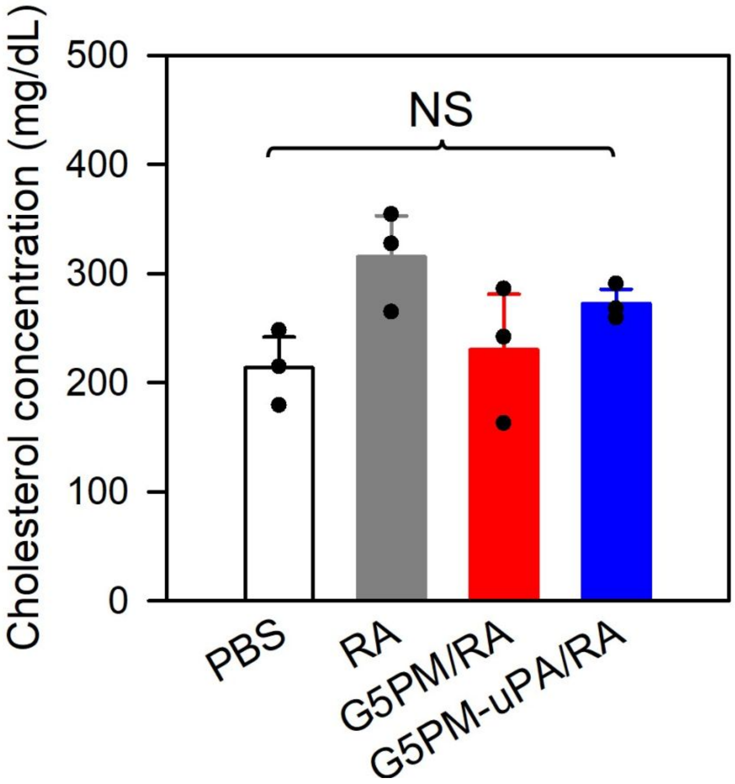

Figure S13. Plasma cholesterol levels did not differ significantly among groups after four weeks of treatment. Data are presented as mean ± SD (n = 3 mice per group; three technical replicates per sample). NS: not significant ($p \geq 0.05$).

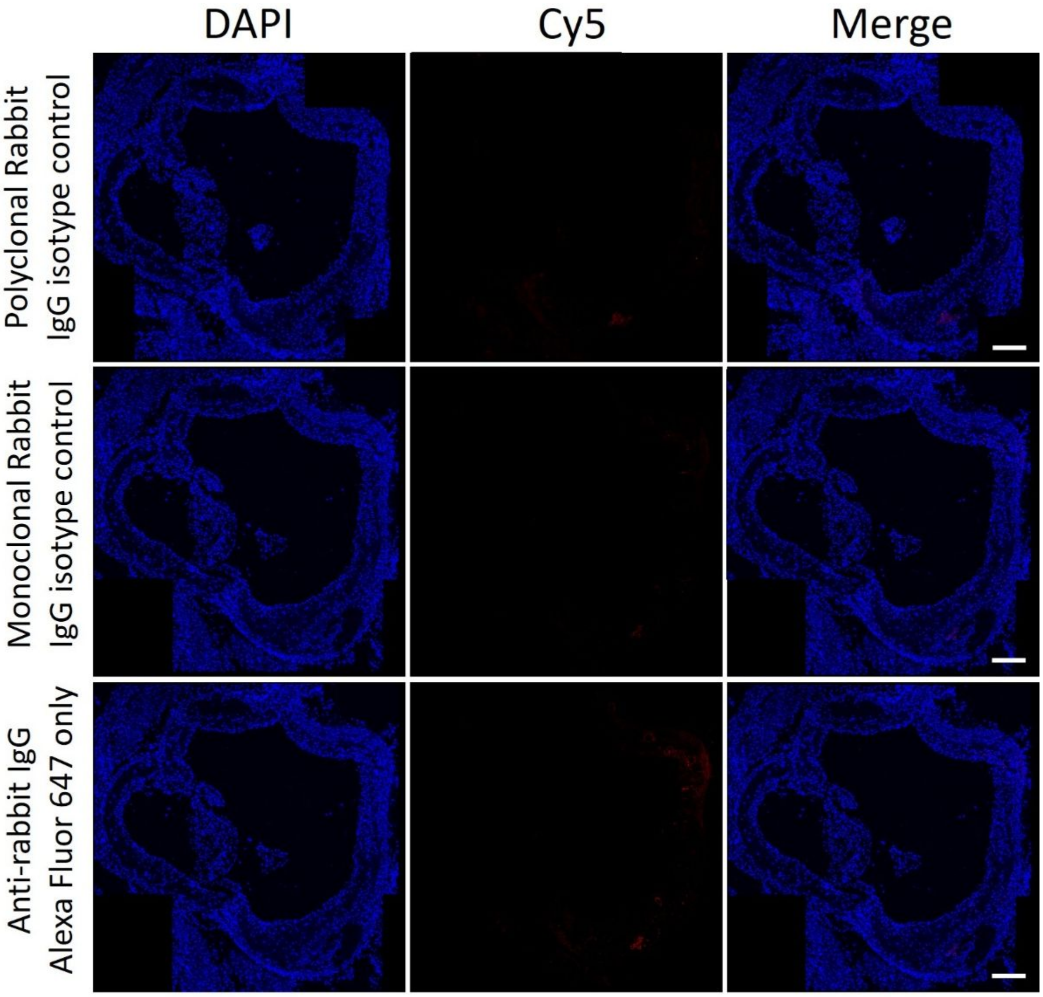

Figure S14. Negative staining controls confirm minimal nonspecific Cy5 signal in aortic root sections. Representative confocal images of aortic root sections stained with Hoechst 33342 (blue) and imaged in the Cy5 channel (red) under three negative-control conditions: polyclonal rabbit IgG isotype control (top row), monoclonal rabbit IgG isotype control (middle row), and secondary antibody only (anti-rabbit IgG Alexa Fluor 647 only; bottom row). Isotype controls were matched to the primary antibody types and used at the same IgG concentration as the corresponding primary. Imaging settings were kept identical to those used for Figure 6. Scale bar: 200 μm.

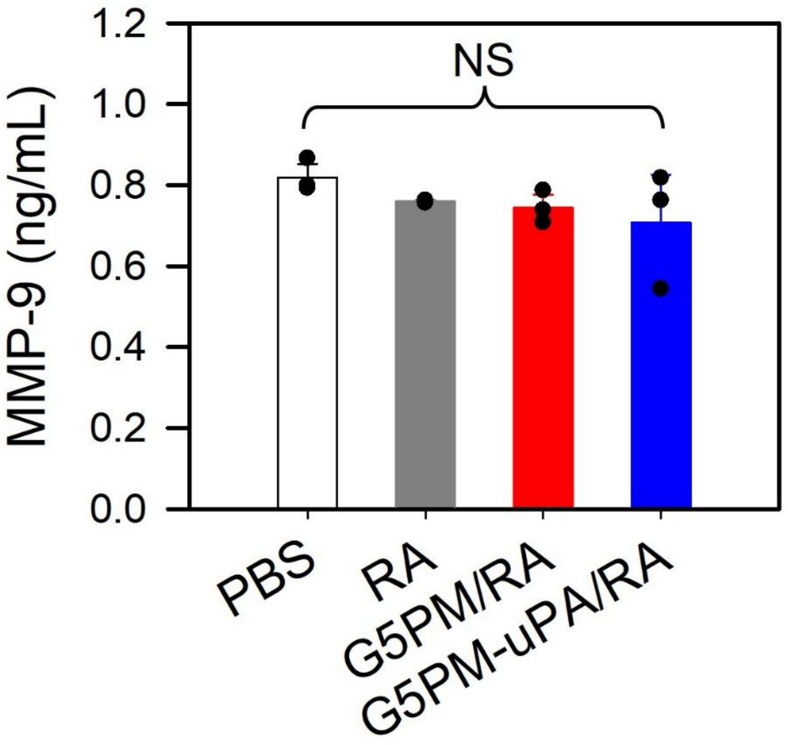

Figure S15. Plasma MMP-9 levels did not differ significantly among groups after four weeks of treatment. Data are presented as mean ± SD (n = 3 mice per group; two technical replicates per sample). NS: not significant ($p \geq 0.05$).

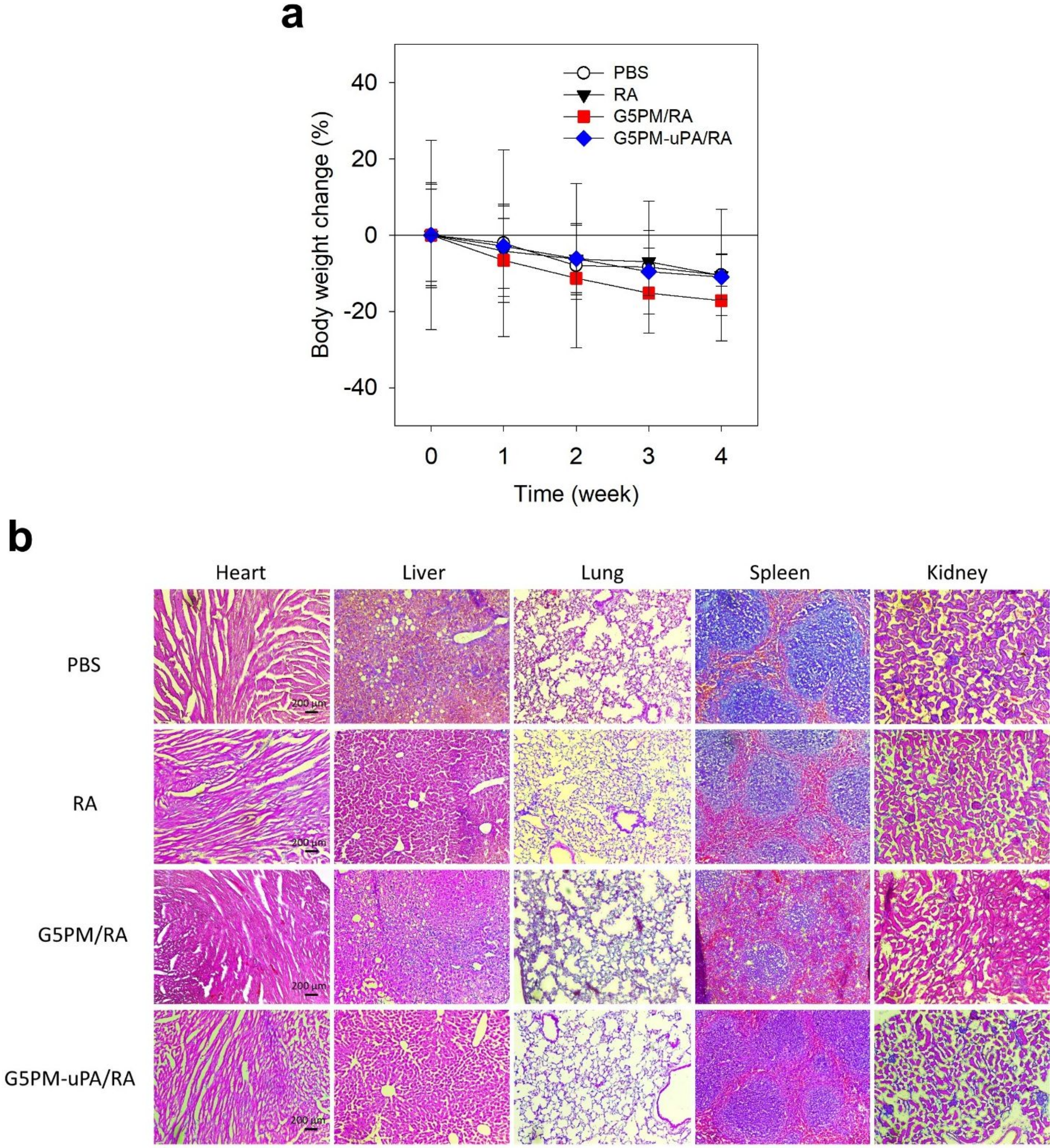

Figure S16. No significant body weight loss nor pathological damage is observed among treatment groups. (**a**) Body weight change percentages (n = 5~8) and (**b**) H&E histological images of main organs from atherosclerosis mice treated with PBS, RA, G5PM/RA, and G5PM-uPA/RA for 4 weeks. Scale bar: 200 µm.

Table S1. Primary and secondary antibodies used in immunofluorescence.

Antibody	Manufacture	Catalog	Titer	MW (kDa)	Host
Anti-TNF-α	Novus Biologicals	NB600587SS	1:500	26	Rabbit
Anti-IL-6	Novus Biologicals	NB6001131SS	1:500	24	Rabbit
Anti-cleaved-caspase 3 (Asp175) (5A1E)	Cell Signaling	9664T	1:500	17, 19	Rabbit
Anti-CD31-FITC	Millipore Sigma	F8402	1:200	N/A	Mouse
Anti-CD68	Invitrogen	FA-11	1:200	N/A	Rat
Anti-α-SMA	Invitrogen	53-9760-82	1:200	N/A	Rat
Anti-rat IgG Alexa Fluor 488	Invitrogen	A-11006	1:500	N/A	Goat
Anti-rabbit IgG Alexa Fluor 647	Invitrogen	A-21245	1:1000	N/A	Goat

Detailed information of the antibodies, including manufacture, catalog, titer, molecular weight (MW) of target protein (kDa), and host. N/A: not applicable.

Table S2. Quantitative summary of histological results.

Treatment	Lesion area in whole aorta (ratio)	Lesion area in aortic root (%)	Necrotic core in aortic root (%)	Fibrous cap in aortic root (%)
Baseline	0.07 ± 0.03	N/A	N/A	N/A
PBS	0.21 ± 0.05	40.7 ± 8.2	38.5 ± 4.6	30.4 ± 7.6
RA	0.16 ± 0.06	39.3 ± 5.8	21.6 ± 6.2	34.9 ± 12.3
G5PM/RA	0.11 ± 0.04	36.5 ± 5.8	18.4 ± 14.7	48.7 ± 10.3
G5PM-uPA/RA	0.10 ± 0.04	24.0 ± 15.3	12.4 ± 6.5	63.5 ± 16.5

Quantitative analysis was performed using ImageJ software (n = 3~4, three sections for each mouse). Data are expressed as mean ± standard deviation (SD). N/A: not applicable.

Table S3. Statistical summary of histological results.

Comparison	*P* value			
	**Lesion area in whole aorta**	**Lesion area in aortic root**	**Necrotic core in aortic root**	**Fibrous cap in aortic root**
Baseline vs. PBS	< 0.001	N/A	N/A	N/A
Baseline vs. RA	0.068	N/A	N/A	N/A
Baseline vs. G5PM/RA	0.698	N/A	N/A	N/A
Baseline vs. G5PM-uPA/RA	0.469	N/A	N/A	N/A
PBS vs. RA	0.459	0.975	0.014	0.888
PBS vs. G5PM/RA	0.009	0.622	< 0.001	0.028
PBS vs. G5PM-uPA/RA	0.015	0.032	< 0.001	< 0.001
RA vs. G5PM/RA	0.545	0.691	0.305	0.139
RA vs. G5PM-uPA/RA	0.696	0.035	0.044	< 0.001
G5PM/RA vs. G5PM-uPA/RA	0.998	0.097	0.298	0.068

Normality and variance were tested using the Shapiro-Wilk and Levene's tests, respectively, and group comparisons used one-way ANOVA (Tukey), Welch's ANOVA (Games-Howell), or Kruskal-Wallis (post hoc) as appropriate. Statistical significance was set at $p < 0.05$ (*$p < 0.05$, **$p < 0.01$, ***$p < 0.001$), and for letter labels, groups sharing any letter are not significantly different while groups with no letters in common are significantly different. N/A: not applicable.

Table S4. **Quantitative summary of immunofluorescence results.**

Treatment	TNF-α (ratio)	IL-6 (ratio)	Cleaved-caspase 3 (ratio)
PBS	1.00 ± 0.34	1.00 ± 0.33	1.00 ± 0.49
RA	0.64 ± 0.51	0.72 ± 0.42	0.50 ± 0.25
G5PM/RA	0.60 ± 0.56	0.37 ± 0.31	0.51 ± 0.27
G5PM-uPA/RA	0.41 ± 0.34	0.43 ± 0.33	0.39 ± 0.22

Mean fluorescence intensity (MFI) for TNF-α, IL-6, and cleaved-caspase 3 was quantified in ImageJ by manually tracing the lesion (plaque) region of interest (ROI) on each aortic root section and measuring Cy5 signal within the traced lesion ROI only. For each marker, values were then normalized to the PBS group and reported as a ratio (PBS = 1.00), where ratios <1 indicate reduced staining relative to PBS. Data are shown as mean ± SD (n = 3~4 mice per group; three sections per mouse).

Table S5. Statistical summary of immunofluorescence results

	P value		
**Comparison**	**TNF-α (%)**	**IL-6 (%)**	**Cleaved-caspase 3 (%)**
PBS vs. RA	0.618	0.409	0.038
PBS vs. G5PM/RA	0.599	0.016	0.047
PBS vs. G5PM-uPA/RA	0.033	0.009	0.001
RA vs. G5PM/RA	1.000	0.299	0.943
RA vs. G5PM-uPA/RA	1.000	0.316	0.303
G5PM/RA vs. G5PM-uPA/RA	1.000	0.986	0.388

Normality and variance were tested using the Shapiro-Wilk and Levene's tests, respectively, and group comparisons used one-way ANOVA (Tukey), Welch's ANOVA (Games-Howell), or Kruskal-Wallis (post hoc) as appropriate. Statistical significance was set at $p < 0.05$ (*$p < 0.05$, **$p < 0.01$, ***$p < 0.001$), and for letter labels, groups sharing any letter are not significantly different while groups with no letters in common are significantly different.
